# Atomistic Simulation of Flow-Induced Microphase Separation and Crystallization of an Entangled Polyethylene Melt Undergoing Uniaxial Elongational Flow and the Role of Kuhn Segment Extension

**DOI:** 10.3390/polym15081831

**Published:** 2023-04-09

**Authors:** Mohammad Hadi Nafar Sefiddashti, Brian J. Edwards, Bamin Khomami

**Affiliations:** Materials Research and Innovation Laboratory, Department of Chemical and Biomolecular Engineering, University of Tennessee, Knoxville, TN 37996, USA; mnafarse@vols.utk.edu (M.H.N.S.); bkhomami@utk.edu (B.K.)

**Keywords:** polyethylene, uniaxial elongational flow, molecular simulation, flow-enhanced nucleation, microphase separation, flow-induced crystallization, Kuhn segment extension

## Abstract

Atomistic simulations of the linear, entangled polyethylene C_1000_H_2002_ melt undergoing steady-state and startup conditions of uniaxial elongational flow (UEF) over a wide range of flow strength were performed using a united-atom model for the atomic interactions between the methylene groups constituting the polymer macromolecules. Rheological, topological, and microstructural properties of these nonequilibrium viscoelastic materials were computed as functions of strain rate, focusing on regions of flow strength where flow-induced phase separation and flow-induced crystallization were evident. Results of the UEF simulations were compared with those of prior simulations of planar elongational flow, which revealed that uniaxial and planar flows exhibited essentially a universal behavior, although over strain rate ranges that were not completely equivalent. At intermediate flow strength, a purely configurational microphase separation was evident that manifested as a bicontinuous phase composed of regions of highly stretched molecules that enmeshed spheroidal domains of relatively coiled chains. At high flow strength, a flow-induced crystallization (FIC) occurred, producing a semicrystalline material possessing a high degree of crystallinity and primarily a monoclinic lattice structure. This FIC phase formed at a temperature (450 K) high above the quiescent melting point (≈400 K) and remained stable after cessation of flow for temperature at or below 435 K. Careful examination of the Kuhn segments constituting the polymer chains revealed that the FIC phase only formed once the Kuhn segments had become essentially fully extended under the UEF flow field. Thermodynamic properties such as the heat of fusion and heat capacity were estimated from the simulations and found to compare favorably with experimental values.

## 1. Introduction

Elongational flows of polymeric liquids are of great theoretical and practical interest. These strong flows can typically induce dramatic changes in the conformation of individual molecules, from random coils in the quiescent state to highly ordered and stretched chains at high deformation rates. The wide variation of conformational changes of individual macromolecules renders a diverse spectrum of complex microstructural and rheological responses that are not trivial to describe theoretically. Examples of such complex responses include extension thinning or thickening, depending on the strain rate [1,2,3,4,5,6], configurational microphase separation of entangled polymer melts at intermediate deformation rates [7,8,9,10], concentration fluctuations [11,12] and chemical phase separation [13,14,15,16] in entangled solutions, and flow-induced melting point elevation [17,18] and crystallization [14,16,17,18,19,20,21,22,23,24,25,26,27,28,29,30,31,32,33,34,35,36] in both melts and solutions.

Sophisticated tube-based constitutive models of entangled liquids that incorporate various relaxation mechanisms, such as reptation, contour length fluctuations, convective constraint release, tube stretch, and finite extensibility of polymer molecules, typically predict qualitatively similar behavior for all entangled solutions and melts in response to elongational flow fields. Specifically, these models predict a nearly constant extensional viscosity, η, within the linear viscoelastic regime, $\dot{\varepsilon }<\tau_{d}^{-1}$, extension thinning within the range $\tau_{d}^{-1}<\dot{\varepsilon }<\tau_{R}^{-1}$, followed by an upturn and extension thickening for $\dot{\varepsilon }>\tau_{R}^{-1}$ driven by chain stretching, and a final plateau-like region at extremely high extension rates where chains become almost fully extended–see, e.g., Figure 1 of Ref. [37]. In these inequalities, $\dot{\varepsilon }$ is the extension rate, $\tau_{d}$ is the disengagement or reptation time of the liquid, and $\tau_{R}$ is the Rouse time. Experimental observations of the rheological responses, however, are not as uniform and unique as the theoretical picture portrays. In fact, in many cases, the tube model fails at predicting the extensional stress and viscosity, even qualitatively, when $\dot{\varepsilon }>\tau_{R}^{-1}$. Specifically, the predicted upturn in elongational viscosity at $\dot{\varepsilon }>\tau_{R}^{-1}$ is not observed in many experimental measurements. Experiments, rather, typically exhibit a monotonic decrease in the extensional viscosity for $\dot{\varepsilon }>\tau_{d}^{-1}$, within the nonlinear viscoelastic regime, even at very high extension rates. Furthermore, the measured viscosity power-law exponents vary over a wide range for different liquids, in contradiction to theoretical predictions.

The discrepancy between theoretical predictions and experimental measurements has been attributed to the different behavior of entangled melts and concentrated solutions in uniaxial flow fields, the argument being that while entangled solutions extension thicken at $\dot{\varepsilon }>\tau_{R}^{-1}$, melt extensional viscosity monotonically decreases in the nonlinear regime [3]. Extension thinning was proposed to be caused by the reduction of monomeric friction at high extension rates as polymer chains became highly oriented and aligned along the flow direction [38,39]. Wingstrand et al. [4] studied the uniaxial extension of entangled solutions of polymethyl methacrylate (PMMA) dissolved in oligomeric (low molecular weight) PMMA and showed that this liquid exhibited a monotonic extension thinning similar to that of entangled polystyrene (PS) melts of a similar number of entanglements per chain. They concluded that using an oligomeric solvent provided a local environment around the polymer molecules analogous to that in a melt, and hence a similar potential for friction reduction that ultimately eliminated the strain hardening of the solution at high extension rates.

Nafar Sefiddashti et al. studied planar elongational flow (PEF) of an entangled linear polyethylene (PE) melt and also an entangled PE solution in n-hexadecane using united-atom nonequilibrium molecular dynamics (NEMD) simulations [7,8,10,16]. They observed monotonic extension thinning for the primary extensional viscosity in the nonlinear viscoelastic regime with power-law exponents in the range −0.35 to −0.4 for $\dot{\varepsilon }>\tau_{R}^{-1}$, in agreement with typical experiments. They also simulated the PEF of an entangled PE solution in benzene as a typical small-molecule organic solvent. They observed a qualitatively similar extension thinning response for $\dot{\varepsilon }>\tau_{R}^{-1}$ for this solution with a scaling exponent of −0.51, contradicting the notion that a small molecule (rather than an oligomeric) solvent exhibits extension hardening at $\dot{\varepsilon }>\tau_{R}^{-1}$. The power-law exponent in the range $\tau_{d}^{-1}<\dot{\varepsilon }<\tau_{R}^{-1}$ was evidently smaller; however, the uncertainty in the calculated viscosities in this region was rather large. Nevertheless, it is not known whether the same response would be observed under uniaxial extension flow (UEF).

Huang et al. [40] examined the extensional viscosity of a series of polystyrene solutions in oligomeric PS at various concentrations but nearly constant number of entanglements per chain. These experiments revealed a wide range of responses from simple extension thinning to substantial thinning at high deformation rates, suggesting that the solvent type (small molecule or oligomeric) is not the only prominent factor of the high extension-rate response. Furthermore, Ianniruberto et al. [6] analyzed experiments on entangled polyisoprene solutions, polyisoprene melts, and poly(n-butyl acrylate) melts, demonstrating the occurrence of strain hardening at high elongation rates and contradicting the notion that all entangled melts monotonically thin under uniaxial elongation.

Overall, experimental evidence implies that there should be a molecular mechanism involved in the high extension-rate response of entangled liquids, which has been overlooked in tube-based models. Ianniruberto et al. [6] proposed that the missing mechanism was the flow-induced reduction of the monomeric friction coefficient due to the alignment of Kuhn segments at high extension rates. They argued that the ratio of the extensional viscosity at low and very high extension rates can be approximated as

(1) $$\frac{3\eta_{0}}{\eta_{\infty }}=\frac{M^{1.4}M_{K}}{M_{c}^{2.4}}\;\frac{\zeta_{\mathrm{eq}}}{\zeta_{\mathrm{aligned}}}\;\;\;,$$

where $\eta_{0}$ and $\eta_{\infty }$ are the zero-shear and asymptotic high extension-rate viscosities, respectively, *M* is polymer molar mass, $M_{K}$ is Kuhn segment molar mass, $M_{c}$ the critical molar mass signifying the onset of the entangled regime, and $\zeta_{\mathrm{eq}}$ and $\zeta_{\mathrm{aligned}}$ are the Kuhn segment friction coefficients under quiescent conditions and extremely high extension rates, respectively.

Nonequilibrium molecular dynamics (NEMD) simulations have been used to examine rheological responses and microstructural properties of polymeric liquids under experimental conditions as well as conditions not conveniently accessible in the laboratory. Understanding the physical mechanisms that govern the flow response of entangled polymers requires an accurate model of the intramolecular and intermolecular interactions that maintains validity over a wide range of length and time scales. Furthermore, a simulation methodology must be able to track the dynamical evolution of the macromolecular constituent particles over the same ranges in length and time scales, including those associated with bond-stretching and bond-bending dynamics up to macroscopic dimensions; therefore, nonequilibrium molecular dynamics is the method of choice for studying the flow dynamics of these complex materials.

Nonequilibrium molecular dynamic simulations were used to study atomic and mesoscale properties under shear and elongational flows, including flow-induced phenomena, over wide ranges of strain rates for a series of unentangled and entangled polymeric liquids during the last few decades [41,42]. Nafar Sefiddashti et al. [7,8] simulated PEF of entangled C_700_H_1402_ and C_1000_H_2002_ monodisperse linear PE melts and examined microstructural (e.g., chain fractional extension), topological (e.g., number of entanglements), and rheological (e.g., viscosity) flow responses with a focus on the nonlinear viscoelastic regime. The response of the C_700_H_1402_ melt was more or less intuitive based on simulation results. Specifically, the steady-state distribution of the chain fractional extension, *x*, was unimodal and Gaussian at equilibrium and low De, where $De\equiv \dot{\varepsilon }\tau_{R}$ is the dimensionless Deborah number. With increasing De, the distribution shifted to higher fractional extensions, demonstrating orientation and stretching of individual molecules in the direction of flow. At high extension rates, e.g., De≈6, molecules were about 80% stretched and fully aligned in the extension direction. Furthermore, the probability distribution function (PDF) of the individual chain fractional extension remained essentially unimodal with a peak position corresponding to the ensemble-averaged fractional extension, 〈x〉. The distributions of the number of entanglement kinks per chain, $Z_{k}$, were also unimodal at all flow strengths (Poisson-like at high De, and Gaussian-like at low and intermediate De). The average number of entanglements decreased with increasing De, in a manner inversely proportional to 〈x〉.

The C_1000_H_2002_ melt behaved very similarly to C_700_H_1402_ undergoing, PEF at low and high De. At intermediate extension rates (i.e., 0.3≤De≤1.5), however, the steady-state PDF of the fractional extension was bimodal with a peak at low values of *x*, corresponding to relatively coiled configurations of a fraction of the chains, and a second peak at relatively high values of *x* corresponding to rather stretched molecules. This behavior was attributed to the coil-stretch transition of the macromolecules [7], qualitatively similar to that predicted for dilute solutions by de Gennes [43]. Furthermore, it was observed that the coiled and stretched molecules underwent a configurational microphase separation such that the coiled molecules segregated into distinct ellipsoidal domains that were surrounded by sheet-like regions of stretched macromolecules. Several other structural and topological properties of the system, including the number of entanglements, the radius of gyration, and the order parameter [17], exhibited similar bimodal distributions with peaks corresponding to the coiled and stretched configurations. These simulations suggested that the existence and manifestation of configurational microphase separation in entangled melts depends on the molecular weight (equivalently, number of entanglements) of the polymeric liquid.

The molecular-weight and strain-rate dependencies of flow-induced microphase separation were examined via high-fidelity nonequilibrium dissipative particle dynamics (DPD) simulations using model parameters that were fine-tuned to capture accurately the dynamics of entangled polyethylene melts [44]. Boudaghi et al. [10] simulated replicas of C_1000_H_2002_ and C_3000_H_6002_ melts (the latter possessing 38 entanglements per chain under quiescent conditions) using a fine-tuned DPD force-field system and demonstrated that the C_3000_H_6002_ melt underwent microphase separation in the range 0.1≤De≤7 in PEF. The biphasic range of C_3000_H_6002_ was significantly wider than that of C_1000_H_2002_ (i.e., 0.3≤De≤3), based on these simulations, verifying the intensifying effect of molecular weight on the strain-rate range where microphase separation was observed. Furthermore, they demonstrated via NEMD and DPD simulations that highly stretched molecules could experience a qualitatively similar configurational microphase separation while relaxing back to the quiescent state after cessation of flow.

In another modeling study, Nafar Sefiddashti et al. [9] expressed the Rolie-Poly constitutive equation [45] in terms of the chain conformation tensor (rather than the stress or tube orientation tensor) and demonstrated that, provided the convective constraint release rate parameter was large enough (i.e., fast disentanglement rates), the model was capable of predicting a bistable steady-state for PEF of entangled liquids at intermediate extension rates. They also investigated the effect of various model parameters and fluid properties on the predicted De range of the bistable region. Different flow-induced inhomogeneities were also observed later in the UEF of shorter normal alkanes [36]. It is worth mentioning that a configurational microphase separation was not reported for concentrated entangled solutions (as opposed to melts); rather, these solutions underwent a thermodynamic phase separation into concentrated domains of polymer and solvent at intermediate and high extension rates; i.e., at De⪆1.5 for entangled solutions of PE in n-hexadecane or benzene solvents [16].

At very high extension rates, some polymeric systems are prone to experience flow-induced crystallization (FIC). This phenomenon has been studied experimentally for several decades [14,19,20,21,22,23,24,25,46] and via nonequilibrium atomistic simulations [16,17,18,26,27,28,29,30,31,32,33,34,35,36]. The majority of the simulation work on FIC followed a protocol consisting of an initial nonequilibrium UEF simulation at temperatures above the melting point of the polymeric liquid, followed by quenching the simulation temperature to a value below the quiescent melting point ranging from 15–35% undercooling. Nafar Sefiddashti et al. studied FIC of a C_1000_H_2002_ PE melt [17] and solution in n-hexadecane [16] under PEF at 450 K, roughly 50 K above the quiescent melting point of linear PE. They demonstrated that a first-order reversible crystallization reaction quantified FIC kinetics fairly accurately, and subsequently calculated the forward and reverse rate constants. The computed values showed that both forward and reverse rate constants scaled linearly with increasing extension rate; however, the scaling slope for the forward rate constant was an order of magnitude greater than that of the reveres reaction, which remained mostly unaffected by strain rate [18].

Due to the relatively long relaxation times of entangled polymers, simulating extensional flows for sufficiently long periods of time requires special periodic boundary conditions (PBCs) to avoid unreasonably small simulation cell dimensions in directions parallel to the compression axes of the elongational flow. This type of PBC was developed by Kraynik and Reinelt, [47] who formulated the KRBC by establishing sufficient conditions for compatibility of arbitrary lattices under PEF. From an experimental perspective, producing PEF in a laboratory is not a simple matter, and the majority of experimental rheological measurements are performed under uniaxial elongation kinematics. Therefore, for PEF, a direct comparison of NEMD results and relatively rare experimental measurements cannot be readily accomplished. However, there is occasional evidence suggesting that the rheological response of entangled liquids under PEF and UEF are similar within a fairly wide range of extension rates. Nguyen et al. [48] modified a filament stretching rheometer and performed PEF and UEF experiments for a 10% solution of polystyrene constituted of 30 entanglements per molecule. These experiments revealed a nearly identical extensional viscosity response for PEF and UEF over the range of extension rates examined; i.e., 0.1<Wi<100, where the Weissenberg number is defined as $Wi\equiv \dot{\varepsilon }\tau_{d}$. Nevertheless, until recently it was not possible to perform sufficiently long UEF simulations to verify similarities in the responses of entangled polymers to different types of elongational flows due to the absence of applicable PBCs.

Boundary conditions were recently developed by Dobson [49] and Hunt [50] for uniaxial elongational flow and implemented into the LAMMPS [51] simulation package by Nicholson and Rutledge [33] to extend UEF NEMD simulation to arbitrarily long times. Murashima et al. [52] performed Kramer/Grest-type coarse-grained NEMD simulations of a lightly entangled (less than three entanglements per chain) melt under planar, uniaxial, and biaxial elongational flows. The extensional viscosities for all three types of extensional flow behaved qualitatively similar at all flow strengths and practically overlapped for $\dot{\varepsilon }>\tau_{R}^{-1}$. Specifically, viscosities exhibited extension thickening in the range $\tau_{d}^{-1}<\dot{\varepsilon }<\tau_{e}^{-1}$, and a plateau region at higher extension rates, where $\tau_{e}$ is the entanglement time. Nevertheless, the lack of extension-thinning behavior in these results is dubious. O’Connor et al. [53] also used a Kramer/Grest bead-spring coarse-grained model in NEMD simulations and studied two series of entangled liquids of various chain stiffness and number of entanglements under UEF. Overall, these simulations suggested that strain hardening became more pronounced as the chain stiffness and number of entanglements decreased. An upturn in extensional viscosity could even disappear for a particular polymer as the chain length (or the number of entanglements) increased. They also underlined the changes in the friction coefficient that occurred due to chain alignment at high flow strength and suggested that macromolecular architecture (e.g., the existence of large side groups attached to the polymer backbone) could significantly alter such changes and, consequently, significantly impact the rheological response at high extension rates.

Nonequilibrium molecular dynamics simulations greatly facilitate the discovery and understanding of a broad spectrum of flow-induced phenomena of entangled polymeric liquids under both shear and extensional flows. While simulating the UEF of these fluids has been made attainable using the Dobson and Hunt (DH) periodic boundary conditions, their computational cost is typically significantly higher than simulating the PEF of a similar liquid. This primarily arises from the necessity of using an initial cubic simulation cell (similar cell dimensions in all three directions of Cartesian coordinates) when employing the DH boundary conditions. On the other hand, the KR boundary conditions only require a rectangular parallelepiped initial cell (with similar cell dimensions in extension and contraction directions). Furthermore, during the course of a simulation, the cell in the DH method does not deviate significantly from its initial cubic shape. Hence, the box length should be chosen long enough such that it can accommodate a substantial portion of a stretched chain at steady-state at the (potentially high) extension rate of simulation. In the KR method, however, the simulation cell length grows in the extension direction between the lattice strain periods (i.e., the period in which the lattice reproduces itself, typically 0.9624 Hencky strain units). This means that the cell size in the extension direction is, on average, significantly (1–2.6 times) longer than its initial length, which in return allows a somewhat shorter initial cell size compared to the DH method. Note that the cell size in the neutral direction of PEF could be as short as the radius of gyration of the polymer under quiescent conditions since no compression or expansion occurs in this dimension. Heuristically, the box volume (or the number of particles) of a PEF simulation of a polymeric liquid using the KRBC would be typically 3–15 times smaller than that of a UEF simulation for a similar liquid using the DHBC. (The average cell length in the extension direction during a lattice strain period ($\varepsilon_{H}=0.9624$) equals 1.68 times the initial cell length in that direction. A cubic box that is 1.68 times longer than the presumed box would have (at least) a $1.68^{3}=4.7$ times larger volume, assuming the KR box size in the neutral direction equals that in the other directions. However, the cell size in the neutral direction could be significantly smaller than the others. Therefore, a 3-times shorter length in the neural direction (as for the PEF of C_1000_H_2002_ in Ref. [8]) leads to a factor 14.2 fewer number of particles). This is exceedingly consequential from a computational cost perspective, especially if the conclusion of Nguyen et al. [48] respecting the equivalency of the PEF and UEF responses of entangled liquids proves to be generally valid. In that event, PEF simulations could be performed at significantly lower computation costs (or, equivalently, run for longer molecules at a similar cost) compared to UEF simulations and still be indirectly compared against experimental data.

An explicit and comprehensive comparison of PEF and UEF responses of entangled liquids, especially at an atomistically detailed resolution, is scarce. Hence the objectives of the present article are two-fold. First, the united-atom NEMD simulation results of uniaxial extensional flow of an entangled linear C_1000_H_2002_ polyethylene melt over a wide range of extension rate are presented, and various structural, topological, rheological, and thermodynamic properties of the system are investigated. Specifically, extension-induced phenomena, including the coil-stretch transition, configurational microphase separation, and flow-induced nucleation and crystallization, as well as their corresponding extension-rate ranges, will be examined systematically. Second, a one-to-one comparison of these results with those obtained for the same PE melt undergoing PEF will be made over the same range of extension rates. The majority of the PEF simulation results presented below were taken from a series of papers of prior work on this subject [7,8,17,18,54].

## 2. Simulation Methodology

Nonequilibrium molecular dynamics simulations of uniaxial elongational flow of the monodisperse linear entangled C_1000_H_2002_ PE melt were performed in the NpT statistical ensemble at 450 K and a density of 0.766 g/cm^3^ (constant pressure corresponding to the equilibrium pressure of the system obtained from equilibrium MD simulations at De=0), spanning a wide range of extension rates, i.e., 0≤De≤60, expressed in terms of a dimensionless Deborah number as defined above. The NpT ensemble was chosen to allow for cell volume and density adjustment, which facilitates the accurate calculation of the structural, rheological, and thermodynamic properties of the system in cases where FIC occurs. The simulation cell contained 540 linear C_1000_H_2002_ macromolecules. At the start of each simulation, the box was cubic of length 254 Å in each direction. As a simulation proceeded, these box dimensions changed as the fluid extended in the flow direction (*z*) and compressed in the orthogonal directions (*x* and *y*). The simulation cell contained 540,000 united atoms, each representing a distinct methyl or methylene unit of the respective chain. Given the enormous computational cost of these simulations, the choice of cell dimensions was motivated based on extensive prior NEMD simulations of the same polymeric melt under planar elongational flow. Steady-state was determined by monitoring key output variables (such as average chain length) and determining that the time evolution of these quantities had been reduced to less than 5% variation.

The NEMD simulations employed the Siepmann-Karaborni-Smit (SKS) united-atom potential [55] to model the energetic interactions between the atoms comprising the C_1000_H_2002_ macromolecules. The SKS model has been used in many prior simulation studies of alkane and polyethylene fluid behavior [41]. However, the rigid bond between adjacent atoms in the original model was replaced with a harmonic potential function [56]; this is a common procedure in molecular dynamics simulations employing this potential model as it alleviates integration difficulties associated with constrained bonds [57,58,59]. The intermolecular and intramolecular nonbonded energetic interactions in the SKS model are given by a 12-6 Lennard-Jones (LJ) potential,

(2) $$U_{LJ}\left(r_{ij}\right)=4\epsilon_{ij}\left[{\left(\frac{\sigma_{ij}}{r_{ij}}\right)}^{12}-{\left(\frac{\sigma_{ij}}{r_{ij}}\right)}^{6}\right]\;\;,$$

where $r_{ij}$ is the distance between atoms *i* and *j*. Note that the intramolecular energy is only applied to atoms separated by more than three bonds. The energetic parameters ($\epsilon /k_{B}$) were 47 K for CH_2_ units and 114 K for CH_3_ units [57,58,59], where $k_{B}$ is Boltzmann’s constant. The distance parameters were $\sigma_{CH_{2}}=\sigma_{CH_{3}}=3.93$ Å for both CH_2_ and CH_3_ units. Lorentz-Berthelot mixing rules were used to calculate the interaction parameters between atomic units *i* and *j*, such that

(3) $$\begin{array}{cc} \epsilon_{ij} & ={\left(\epsilon_{i}\epsilon_{j}\right)}^{1/2}\;\;,\\ \sigma_{ij} & =\frac{\sigma_{i}+\sigma_{j}}{2}\;\;. \end{array}$$

A cutoff distance $r_{c}=2.5\sigma$_CH_2__ was employed for all LJ potentials.

The bond-stretching interaction energy was described by a harmonic potential function,

(4) $$U_{str}\left(l\right)=\frac{1}{2}k_{l}{\left(l-l_{eq}\right)}^{2}\;\;,$$

where *l* is the distance between adjacent atom centers of the same molecule. The bond-stretching constant was $k_{l}/k_{B}=452,900$ K/Å^2^ and the equilibrium bond length was $l_{eq}=1.54$ Å for adjacent united-atom particles. The bond-bending potential energy also was governed by a harmonic potential function of the form

(5) $$U_{ben}\left(\theta \right)=\frac{1}{2}k_{\theta }{\left(\theta -\theta_{eq}\right)}^{2}\;\;,$$

where θ is the angle formed between three successive atoms. The bond-bending constant in this equation was $k_{\theta }/k_{B}=62,500$ K/rad^2^ and the equilibrium angle was $\theta_{eq}=114^{\circ }$.

The bond-torsional interaction energy was expressed as

(6) $$U_{tor}\left(\phi \right)=\sum_{m=0}^{3}a_{m}{\left(\cos\phi \right)}^{m}\;\;,$$

where the bond-torsional coefficients were $a_{0}/k_{B}=1010$ K, $a_{1}/k_{B}=-2019$ K, $a_{2}/k_{B}=136.4$ K, and $a_{3}/k_{B}=3165$ K. This potential was used on atoms of the same chain that were separated by three bonds. Using the physical parameters described above, the maximum possible extension of the C_1000_H_2002_ chains is approximately 1290 Å when all bond dihedral angles assume *trans* configurations, with all bonds and angles taken to be at their equilibrium values.

NEMD simulations of uniaxial elongational flow were performed in the *NpT* statistical ensemble using the Nosé-Hoover thermostat and barostat [60,61,62] implemented within the Large-scale Atomic/Molecular Massively Parallel Simulator (LAMMPS [51]) computing environment developed at Sandia National Laboratory, subject to the UEF velocity gradient tensor,

(7) $$\nabla u=\left(\begin{array}{ccc} -\dot{\varepsilon }/2 & 0 & 0\\ 0 & -\dot{\varepsilon }/2 & 0\\ 0 & 0 & \dot{\varepsilon } \end{array}\right)\;\;$$

Boundary conditions were periodic at all box surfaces with a deforming/rotating simulation box that elongated in the *z* direction and compressed in the *x* and *y* directions. The NEMD equations of motion were integrated using the reversible-Reference System Propagator Algorithm, r-RESPA, with two different time steps: a long time step of 4.7 fs, which was used for the slowly varying nonbonded LJ interactions, and a short time step of 1.176 fs (one-fourth of the long time step) for the rapidly varying forces, including bond-bending, bond-stretching, and bond-torsional interactions. The relaxation time of the thermostat and barostat were set equal to 100 and 10 times the long time step, respectively. The barostat was applied to the contracting *x* and *y* dimensions to maintain a constant pressure equal to the equilibrium pressure of the system in those directions. This was especially useful when FIC occurred and a denser semicrystalline phase formed within the simulation cell: in an NpT simulation, the cell size and volume can be adjusted accordingly to capture the new density of the semicrystalline polymer. Note that a constant pressure in the contraction directions corresponded to free surfaces in those directions, while the fluid was stretched along the other perpendicular direction according to the uniaxial elongational deformation. Simulations were started from an equilibrated configuration at De=0 for a specific De (≠0) and run until all statistical indicators revealed that a steady-state condition had been achieved. The Dobson-Hunt (DH) [49,50] periodic boundary condition was employed to allow for unrestricted simulation time of the deforming simulation box undergoing uniaxial elongational flow over the full range of imposed strain rates.

Table 1 displays equilibrium properties of the simulated melt under quiescent conditions as obtained from equilibrium MD simulations. These properties are the same as indicated in prior work of this particular simulated melt [63,64]. The reptation or disengagement time, $\tau_{d}$, was obtained from the decorrelation time of the chain end-to-end unit vector autocorrelation function [63,65,66,67], and the Rouse time, $\tau_{R}$, was calculated as $\tau_{R}=\tau_{d}/3\langle Z\rangle$ according to reptation theory [68]. ${\langle R^{2}\rangle }^{1/2}$ and ${\langle R_{g}^{2}\rangle }^{1/2}$ represent the ensemble-averaged chain end-to-end distance and the radius of gyration, respectively. The ensemble-averaged number of entanglements, 〈Z〉, and kinks, $\langle Z_{k}\rangle$, (each on a per-chain basis) were calculated using the Z1 code developed by Kröger [69,70].

Theoretically, one can calculate the equilibrium chain entanglement number by $\langle Z\rangle ={\langle L\rangle }^{2}/\langle R^{2}\rangle$, where 〈L〉 is the average primitive chain contour length. The entanglement network topological analysis using the Z1 code provides 〈L〉 (508.6 Å) for calculation of 〈Z〉 (12.9). The effective reptation tube diameter, $a_{t}$, can be obtained from the relationship $a_{t}=\langle L\rangle /\langle Z\rangle$ as 39.4 Å. Moreover, the Z1 code introduces an alternative topological quantifier, the number of kinks per chain, $\langle Z_{k}\rangle$. Typically, the number of kinks is roughly twice the number of entanglements under quiescent conditions; however, it is unknown if such a relationship applies away from the equilibrium state. It is also not clear if the physical definitions of 〈Z〉, 〈L〉, and $a_{t}$ remain valid under nonequilibrium conditions. Therefore, in the following, $\langle Z_{k}\rangle$ is used primarily as the indicator of entanglements under nonequilibrium flow conditions since it is directly calculated by the Z1 code via an unambiguous geometrical algorithm.

According to equilibrium (De=0) simulations, the Kuhn length is estimated as $L_{K}=\langle R^{2}\rangle /{\left|R\right|}_{\max}=15.6$ Å, which implies $N_{K}={\left|R\right|}_{\max}/L_{K}=83$ Kuhn segments. Consequently, an estimate of the equilibrium ${\langle R^{2}\rangle }^{1/2}={\left(N_{k}L_{k}^{2}\right)}^{1/2}=142.1$ Å demonstrates internal consistency when compared to the tabulated value of 141.8 Å. Note that each Kuhn segment is constituted by 12 carbon atom units. According to $\langle R_{g}^{2}\rangle =N_{K}L_{K}^{2}/6$, a theoretical estimate of ${\langle R_{g}^{2}\rangle }^{1/2}$ is thus 58.0 Å, which closely matches the simulated value of 57.9 Å displayed in Table 1. The entanglement molecular weight for linear polyethylene at 443 K of Me=1150 g/mol was reported by Fetters et al. [71], which can be used to estimate an experimental entanglement density of 〈Z〉=M/Me=12.2 and tube diameter $a_{t}={\langle R^{2}\rangle }^{1/2}/{\langle Z\rangle }^{1/2}=40.7$ Å. These values agree well with the simulation results.

## 3. Results and Discussion

### 3.1. Microstructural and Rheological Properties

Polymer dynamics are characterized by a wide range of length and time scales, ranging from Angstroms to meters and femtoseconds to years, respectively. Processing operations span many orders of magnitude of time and length scales in virtually all applications. Different flow strengths and types lead to various flow-induced phenomena and rheological responses in polymeric liquids that are each best scrutinized with special microstructural quantities, depending on the time and length scales inherent to the specific phenomenon under investigation. These quantities range from long length and time scale properties, such as chain end-to-end distance and radius of gyration that influence mechanical properties like the rheological characteristic functions, to small length and time scale properties, such as the local order parameter and atomistic thermodynamic-like variables [18] relevant to flow-induced crystallization.

#### 3.1.1. Configurational Microphase Separation and General Flow Behavior

**Figure 1:**
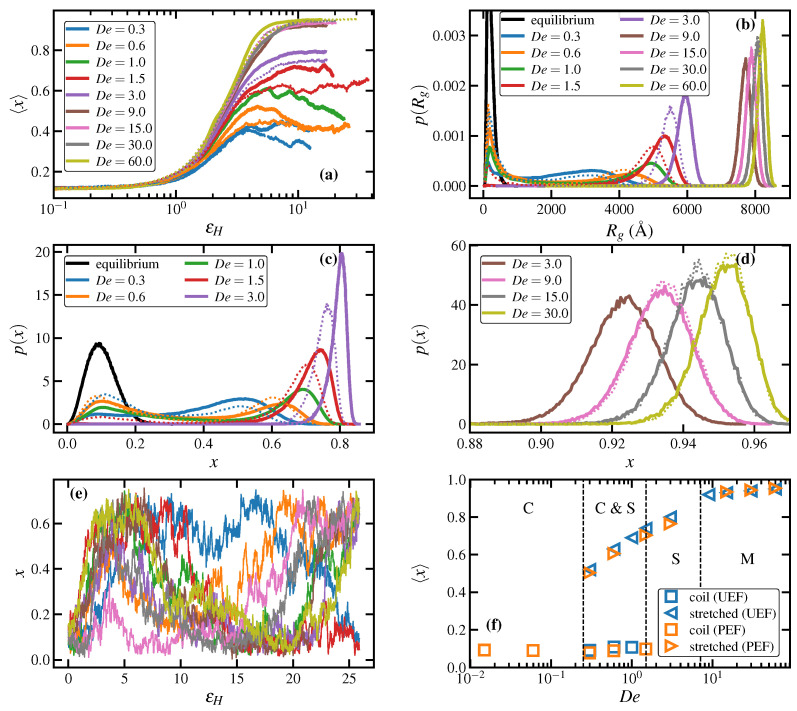
(**a**) Mean fractional extension, 〈x〉, as a function of Hencky strain, $\varepsilon_{H}$, for startup of elongational flow at various values of De: solid lines correspond to UEF and dotted lines to PEF. (**b**) Steady-state probability distribution functions, $p(R_{g})$, of the molecular radius of gyration, $R_{g}$, at various values of De in both UEF and PEF. Panels (**c**,**d**) display steady-state probability distribution functions, p(x), of the fractional extension at various values of De; (**c**) displays p(x) at lower De whereas (**d**) presents p(x) at high De where FIC was observed. (**e**) Fractional extension vs. $\varepsilon_{H}$ for several selected individual chains undergoing startup of UEF at De=0.6 depicting the gradual transitioning of molecules between coiled and stretched configurations. (**f**) Steady-state mean fractional extension versus De for UEF and PEF; blue symbols denote UEF data and orange symbols denote data taken from PEF simulations. Vertical dashed lines delineate approximate boundaries of various configurational states or phases: C, S, and M denote coiled liquid, stretched liquid, and predominantly monoclinic semicrystalline states, respectively.

The mean fractional extension of chains, $\langle x\rangle \equiv {\langle R^{2}\rangle }^{1/2}/{\left|R\right|}_{\max}$, is defined as the ensemble-averaged end-to-end distance normalized by the theoretical maximum end-to-end distance ${\left|R\right|}_{\max}$ (=1290 Å for $C_{1000}H_{2002}$). Figure 1a displays the average fractional extension as a function of the Hencky strain, $\varepsilon_{H}=\dot{\varepsilon }t$, for startup of uniaxial elongational flow (solid lines) at various values of De. Flows of De<0.3 were not simulated (except for the quiescent case of De=0) because of the excessive computational resources required for such large simulation cells in UEF at very low De and because the results of the prior PEF simulations [7,8,16,17] revealed that only the expected behavior arising from reptation theory manifested at such small strain rates. For comparison, the values obtained from prior PEF simulations are also presented in Figure 1a using dotted lines of the same color for similar values of De. Note that PEF simulations at De=1 and De=9 were not performed in the prior work due to limitations of computational resources [7,8,16,17]; hence these two De values for PEF are not included in Figure 1 and other figures throughout the paper.

The mean fractional extension increases very slowly at all De during the first Hencky strain of deformation. At higher strains, 〈x〉 begins to grow at a faster rate with a slope that increases with De. The transient response of 〈x〉 afterward can be classified into three categories of extension rates, namely, 0.3≤De<1.5, 1.5≤De<9, and De≥9. Simulations of UEF at De<0.3 were not feasible from a computational standpoint, but results are expected to be very similar to those of PEF for De<0.3 [7,8,17,18]. In the first region, 0.3≤De<1.5, the fractional extension undergoes an overshoot roughly at $\varepsilon_{H}\approx 4$–5.5, followed by a gradual decay to an eventual quasisteady-state plateau. However, the mean fractional extension at $\varepsilon_{H}>6$ fluctuates such that an indisputable steady state cannot be achieved even after $\varepsilon_{H}\approx 20$ units of deformation. This leads to some ambiguity in determining the steady-state fractional extension. Indeed, the steady-state fractional extension in this region could change significantly depending on the cutoff Hencky strain for calculating the ensemble averages. As discussed below (with reference to Panel (e) of Figure 1), this is caused by individual macromolecules gradually transitioning from relatively coiled to more stretched configurations, and vice versa. Consequently, steady-state values throughout this article were calculated as ensemble averages of the respective data at $\varepsilon_{H}\geq 8$. The lower limit Hencky strain of 8 was chosen because this value is roughly the highest deformation that can be achieved using common rheological measurements of polymeric liquids for reporting steady-state properties, such as extensional viscosity. The fractional extension exhibited in the UEF simulations at these De is qualitatively similar to that of PEF, although quantitatively there is a greater degree of extension in the UEF simulations due to the higher degree of stretching in UEF imparted by the compression in both directions perpendicular to the direction of flow.

In the second region, 1.5≤De<9, the overshoot in mean fractional extension vanishes, and 〈x〉 smoothly approaches its steady-state value at $\varepsilon_{H}\approx 8$. Unlike the lower De range, the fluctuation in 〈x〉 at steady state is insignificant in this range of extension rates, indicating that individual macromolecules mostly remain in either relatively stretched or coiled configurations. Furthermore, the steady-state fractional extension monotonically increases as De increases.

The behavior of 〈x〉 is fairly similar at higher extension rates in the third region, i.e., De≥9, except that the steady-state 〈x〉 exhibits a jump compared to the second region, resembling a discontinuity in steady-state fractional extension, and remains nearly independent of De. This discontinuity is associated with flow-induced crystallization, which will be discussed later. The fractional extension under PEF is qualitatively and quantitatively similar to that of UEF within this range of De. Furthermore, at De=9.0, which is at the lower limit of the FIC range, the initial increase of 〈x〉 with $\varepsilon_{H}$ is commensurate with that of lower values of De. However, at $\varepsilon_{H}\approx 4$, it experiences an increase in slope before settling at its steady-state value at $\varepsilon_{H}\approx 6$. Hence the initial response at De=9.0 is that of a stretched liquid state, which begins to evidence FIC only once the molecules are sufficiently extended at $\varepsilon_{H}\approx 4$.

The steady-state probability distribution functions (PDFs) for the molecular radius of gyration, $p(R_{g})$, and fractional extension, p(x), are plotted in Figure 1b–d for both UEF and PEF. Under quiescent conditions, the PDFs depict essentially coiled macromolecules distributed almost symmetrically about the peak values (i.e., possessing Gaussian-like characteristics), with means of 57.9 Å and 141.8 Å, respectively, as indicated in Table 1. The three characteristic extension rate regions described above can also be recognized in these PDF plots. It is evident from these distributions that the flow response in the first regime, 0.3≤De<1.5, is quite different and somewhat unexpected compared to the other two flow strength regimes. Specifically, the PDFs are broad and bimodal, with one peak positioned at values approximately corresponding to the radius of gyration and end-to-end distance of the molecules under quiescent conditions, mostly assuming coiled configurations, and a second peak appearing at relatively high values of $R_{g}$ and *x*, corresponding to more highly stretched configurations. Note that the coiled-peak position is somewhat independent of flow strength, whereas the stretched-peak moves to the right (higher chain extension) as De increases. This behavior was attributed to a coil-stretch transition in entangled melts, as reported in the past for PEF simulations of $C_{1000}H_{2002}$ and $C_{3000}H_{6002}$ PE melts using NEMD [7] and DPD [10]. In this range of flow strength, the chain-like macromolecules can exist in either of the two stable configurational states (i.e., coiled and stretched). Moreover, individual chains can occasionally transition from one configurational state to the other. Such transitions may be inferred from the small but nonzero population of the chains between the two peaks of the distributions—see the orange curves (De=0.6) of Figure 1b,c, for instance.

Figure 1e depicts the transition of a few selected molecules between the coiled and stretched states at De=0.6 by plotting their individual chain fractional extension as a function of Hencky strain. These plots indicate that the transitioning of molecules between the two states is a random and gradual process, taking about 10 Hencky stain units of deformation (or dimensionless time). Such random exchanges between the coiled and stretched populations give rise to significant fluctuations in the mean fractional extension of the chains, as discussed earlier—see the blue, orange, and green curves of Figure 1a corresponding to De=0.3,0.6,1.0, respectively. These fluctuations of individual chain extension could manifest as fluctuations (or even anomalies) in other properties of the system, such as stress and extensional viscosity, which will be discussed later. Both UEF and PEF simulations exhibit similar behavior, although the stretched-peaks of UEF are narrower and shifted to higher extensions compared to those of PEF.

In the second region, 1.5≤De<9, the distributions p(x) and $p(R_{g})$ become unimodal and sharply peaked, shifting to the right as De is increased, implying a highly stretched liquid state at higher extension rates. The PDFs remain unimodal at even higher extension rates, i.e., De≥9; however, the peak positions shift discontinuously to higher extension at De=9 and asymptotically approach that of fully stretched molecules (x→1). The observed discontinuity resembles a first-order phase transition, and it can be associated with flow-induced crystallization, as briefly mentioned earlier and discussed in Section 3.1.2 in greater detail. Note that the UEF and PEF distributions at De≥9 are virtually identical to each other.

A rough sketch of a configurational phase diagram can be constructed by plotting the peak positions of the steady-state p(x) at various De, as shown in Figure 1f. Blue symbols denote UEF data, whereas orange symbols specify PEF data. Furthermore, squares represent coiled configurational states and triangles denote stretched states. These data points were obtained from the positions of the relevant peaks of the p(x) distributions, as displayed in Figure 1c,d. At low De<0.3 (PEF data only), the melt is composed of coiled molecules that are barely discernible from those of the quiescent state. At 0.3≤De<1.5, the liquid is comprised of molecules that are relatively coiled and others that are relatively extended. At 1.5≤De<9, the steady-state liquid is comprised solely of relatively extended molecules, whereas at De≥9 one observes a predominantly monoclinic flow-induced semicrystalline state.

**Figure 2:**
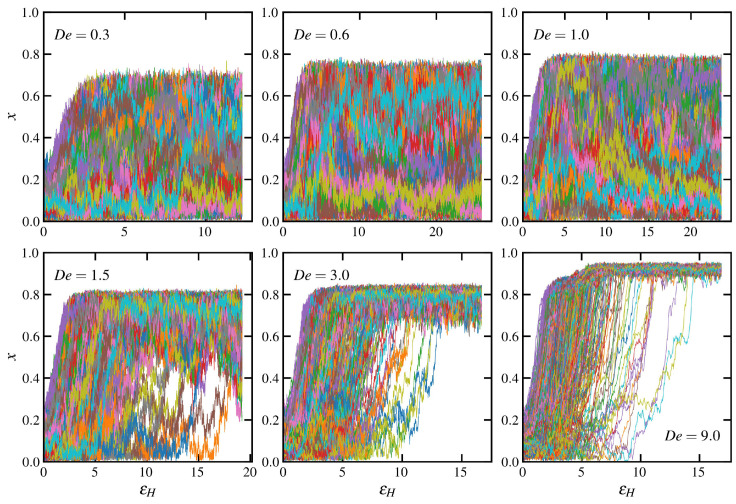
Time evolution of fractional extension of all molecules after startup of UEF at various De plotted in terms of Hencky strain, $\varepsilon_{H}$. Each individual molecular trajectory is plotted using a line of randomly chosen color.

Individual molecular trajectories of fractional extension are plotted vs. Hencky strain in Figure 2 for startup of UEF at various De. At De=0.3,0.6,1.0, the distribution of individual molecular configurations is quite wide, as indicated by the broad PDFs of Figure 1a. There are molecules that rapidly become relatively highly extended upon application of flow, and others that remain relatively coiled even after 20 Hencky strain units. Other molecules gradually transition from one configurational state to the other over multiple $\varepsilon_{H}$ units. (This is hard to visualize from Figure 2 because of the large number of molecules in the UEF simulations; see Figure 1e for an easier visualization of some selected chains). At De=1.5,3.0, all macromolecules eventually assume relatively stretched configurations, although some molecules rapidly increase in extension after inception of flow, whereas others slowly transition from a relatively coiled configuration over a much longer period of time. At De=9, many molecules rapidly rise to a pseudosteady state at $\varepsilon_{H}\approx 3$, corresponding to a stretched liquid state, and then quickly transition to an even more extended state at $\varepsilon_{H}\approx 6$, which is a manifestation of FIC. Even so, there remain some relatively coiled molecules that only begin to transition to the stretched state after $\varepsilon_{H}\approx 10$.

**Figure 3:**
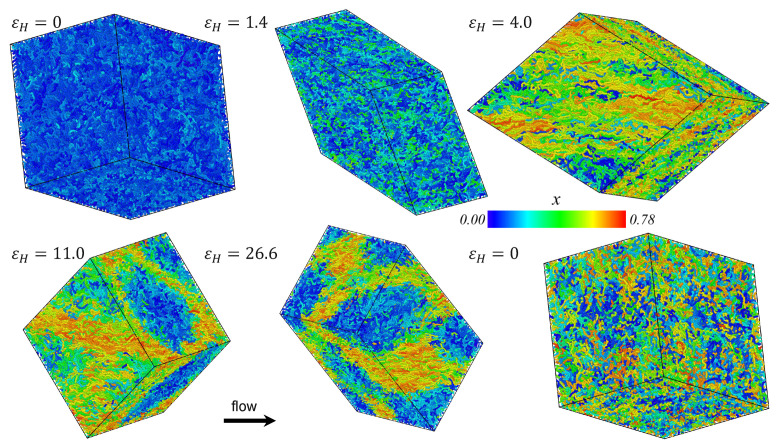
Transient snapshots at various values of Hencky strain of the PE melt undergoing startup of UEF at De = 0.6. Individual macromolecules are assigned a color based on their fractional extension: cooler colors denote relatively coiled chains whereas warmer colors imply stretched chains. The bottom right snapshot is the same as the upper left snapshot corresponding to $\varepsilon_{H}=0$, except that the colors of the individual molecules are assigned according to the final steady-state extension, not that which they possessed at t=0. Note that the boxes are displayed at random angles (and sizes) relative to the flow direction because the simulation cell rotates (and elongates/compresses) with time under UEF due to the imposed boundary conditions.

Snapshots of the simulation cell of the PE melt undergoing startup of UEF at De=0.6 are displayed in Figure 3 at various values of Hencky strain. Cooler colors are assigned to molecules that are relatively coiled whereas warmer colors denote relatively stretched molecules. Under quiescent conditions ($\varepsilon_{H}=0$), the molecules are exclusively coiled. With the onset of flow, certain individual molecules begin to elongate in the direction of flow, whereas others remain relatively coiled, retaining configurations that are essentially similar to those they possessed under quiescent conditions. As time elapses, the stretched molecules approach steady-state configurations and the coiled molecules mostly remain unaffected by the flow; however, after a substantial amount of deformation has occurred, a bicontinuous phase structure is developed wherein the stretched chains form into a mesh-like network of highly elongated molecules that enwrap ellipsoidal domains of relatively coiled chains. The bicontinuous inhomogeneous phase appears to be formed by a migration of stretched and coiled chains into domains of similarly configured molecules rather than a spontaneous heterogeneous development at certain locations within the simulation cell. This statement is supported by the lower right snapshot, which is the same snapshot at $\varepsilon_{H}=0$ but with the assignment of individual molecular colors according to the final steady-state configuration rather than the corresponding one at t=0; this snapshot provides evidence that the chains forming specific domains at steady-state are more or less randomly distributed throughout the simulation cell at t=0.

The spatial inhomogeneity of the biphasic structure exhibited under UEF is distinctly different than that exhibited for the same PE melt undergoing PEF, as presented in preceding publications [8,10,17]. In PEF, a biphasic system is also formed over a similar range of De, but there the heterogeneous liquid is comprised of sheet-like regions of highly stretched molecules that interweave between spheroidal domains of relatively coiled molecules. This difference seems intuitively to be a manifestation of the fact that PEF maintains a neutral direction, whereas UEF implies compression in both directions perpendicular to that of flow. It is not clear, in either PEF or UEF, if the coiled chains are migrating to regions near the flow stagnation point, or vice versa; at first glance, this does not appear to be the case, but the nature of applying the KRBC or DHBC complicates the analysis considerably.

**Figure 4:**
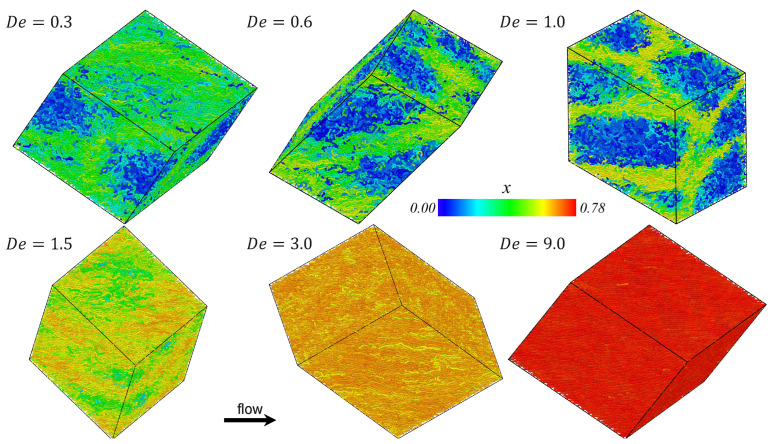
Snapshots of steady-state UEF of the PE melt at various values of De. Cooler colors denote relatively coiled molecules whereas warmer colors signify more highly stretched molecules. The black arrow denotes the direction of flow for all snapshots.

Snapshots of the PE melt undergoing steady-state UEF at various De are displayed in Figure 4. Individual chain molecules are again colored according to the relative end-to-end extension, with tightly coiled chains displayed in a cool blue color and highly extended chains in a warm red color. The bicontinuous liquid microstructure is observed throughout the range 0.3≤De<1.5, where a 3-dimensional mesh-work of elongated chains essentially envelope spatial domains of concentrated coiled molecules. At lower De, the numbers of molecules comprising the two phases are similar (see Figure 2), but as De increases, the fraction of coiled chains diminishes relative to the fraction of stretched chains. However, even at De=1.5, vestigial traces can be discerned of a spatially inhomogeneous bicontinuous liquid phase (see the bottom left snapshot of Figure 4), although the difference in extension of the coiled and stretched phase molecules is rather slight and the p(x) distribution of Figure 1 remains practically unimodal. At higher De, the inhomogeneous microstructural has essentially dissolved into a homogeneous liquid phase composed of highly stretched molecules.

As discussed briefly above, Figure 1f displays the average steady-state fractional extension for different states of the liquid as a function of De for the UEF and PEF simulations. Hence, in the range 0.3≤De<1.5, where the distributions are bimodal, two values are plotted corresponding to the coiled and stretched states. For other flow strengths (De≥3.0), where p(x) is unimodal, the peak position is virtually the same as the mean fractional extension of the highly stretched liquid. Note that PEF and UEF data are practically the same, indicating that this biphasic microstructure is possibly a universal phenomenon of elongational flows. Due to the configurational bistability of the system at intermediate extension rates, Figure 1f effectively exhibits a hysteresis loop in the fractional extension profile of the liquid at steady state. It should be noted that exhibiting a hysteresis means that either purely coiled or stretched states within the bistable region should be achievable by some appropriate manipulation of the flow kinematics. This hysteresis phenomenon has not been directly studied herein; i.e., the flow conditions under which a coiled-only or stretched-only configurational state could appear in the range 0.3≤De<3.0. However, it was demonstrated in previous work [7,10] on PEF of $C_{1000}H_{2002}$ that a metastable stretched-only state could be attained by decreasing the flow strength of a system composed of stretched molecules at steady state at a higher value of De (e.g., De=3) to a lower De located within the bistable region (e.g., De=0.6). In other words, the purely stretched state of the bistable region could be generated by moving from the right branch (high De) of the sigmoidal curve in Figure 1f toward the left (low De). Nevertheless, it was also demonstrated that such purely stretched states were not globally stable and eventually transitioned into biphasic states similar to those depicted in Figure 3 and Figure 4 for sufficiently long simulation times [7,10].

As noted in prior simulations of the same PE melt undergoing steady-state PEF [8], the extensional stress and temperature of the bicontinuous phase remain homogeneous throughout the simulation cell—see Supplementary Materials of Ref. [8]. This implies that the average stress state is not affected by spatial location within the simulation cell, indicating a homogeneous stress state in both space and time. Consequently, the segregation of the macromolecules observed does not seem to be driven by stresses developed within the system under UEF or PEF. This observation points to a microphase separation that is thermodynamically induced by architecturally identical chain molecules whose sole point of difference lies in their adopted individual steady-state configurations which are developed under flow.

**Figure 5:**
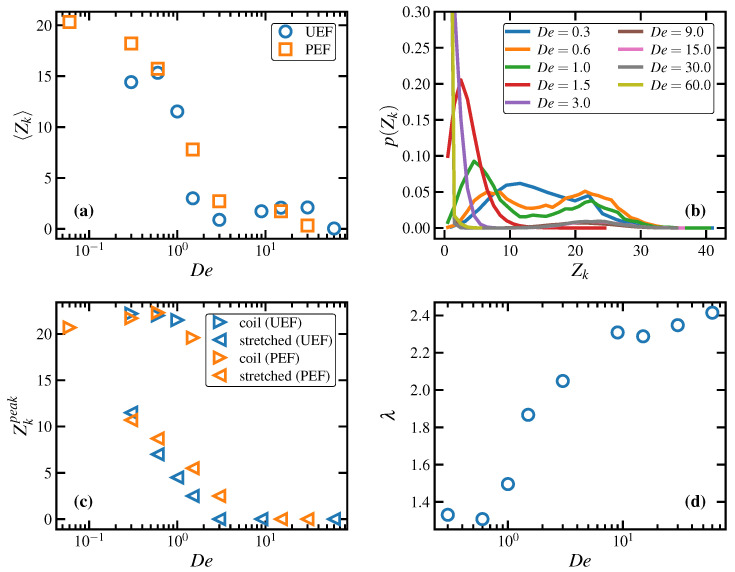
Steady-state topological characteristics of the $C_{1000}H_{2002}$ melt undergoing UEF and PEF: (**a**) average number of chain kinks per molecule vs. De; (**b**) probability distributions of $Z_{k}$ at various De (UEF only); (**c**) peak positions of the PDFs of panel (**b**) plotted vs. De, including those of the same melt undergoing PEF (which are not shown in panel (**b**); and (**d**) tube stretch vs. De under UEF only. Note that the PDFs of De≥9.0 in panel (**b**) all overlap each other.

Topological properties of the $C_{1000}H_{2002}$ melt are displayed in Figure 5 for both UEF and PEF at various De as calculated using the Z1 code [69,70]. Figure 5a displays the ensemble-averaged number of kinks per chain, $\langle Z_{k}\rangle$, plotted as a function of De under both types of flow. At low De (<0.3; PEF only), $\langle Z_{k}\rangle$ remains close to its quiescent value of 24.3, roughly twice the number of entanglements. At intermediate De within the biphasic region (0.3≤De<1.5), however, $\langle Z_{k}\rangle$ drops precipitously to a low value less than 2.5, which subsequently remains relatively close to zero at all higher De.

Figure 5b displays the steady-state PDFs of the number of entanglement kinks, $p(Z_{k})$, of individual chains at various De for the $C_{1000}H_{2002}$ melt undergoing UEF. Within the biphasic region, these PDFs are decidedly bimodal, with peaks located at both small and large values of $Z_{k}$. The high-$Z_{k}$ peak is associated with the coiled liquid phase, whereas the low-$Z_{k}$ peak is associated with the stretched phase. Indeed, both phases tend to maintain the same level of entanglements that would be expected from either a homogeneous coiled or stretched liquid phase hypothetically existing under the same flow conditions. At higher De where only the homogeneous stretched liquid state exists (3.0≤De<9.0) as well as the FIC phase (De≥9.0), the $p(Z_{k})$ are unimodal, possessing tall, narrow peaks at very small values of $Z_{k}$, indicating almost completely unentangled systems. (Note that the PDFs of the FIC phases all overlap each other in the plot).

The positions of the peaks, $Z_{k}^{peak}$, of the PDFs displayed in Figure 5b (as well as those of the corresponding PDFs under PEF; not shown) are plotted versus De in Figure 5c. This entanglement phase diagram makes clear the biphasic nature of the intermediate De (0.3–1.0) regime with its characteristic inverse-S shape, wherein higher values of $Z_{k}$ are associated with the coiled phase and lower values with the stretched phase. It also implies that metastable entanglement phases of either homogeneous coiled or stretched states could be induced by gradually increasing or decreasing De from a monophasic De state point, leading to a hysteresis effect; such stretched metastable states were previously observed for the same $C_{1000}H_{2002}$ melt under PEF at De=0.6,1.5 [17].

The tube stretch is plotted versus De for the $C_{1000}H_{2002}$ melt under steady-state UEF in Figure 5d. At low De, the tube stretch increases only slightly from its quiescent value of unity, and this trend continues for De as high as 0.6, which is well within the nonlinear viscoelastic regime. At De=1, which corresponds to the applied strain rate, $\dot{\varepsilon }$, equalling the reciprocal of the Rouse time, $\tau_{R}^{-1}$, the tube stretch parameter begins to climb substantially with De, indicating strong stretching of the polymer entanglement strands. This increase in chain extension is commensurate with the decrease in chain entanglements discussed above. The rate of increase of λ with De eventually plateaus for De≥9.0, where FIC has evidently occurred. Note that the plateau value of ≈2.4 observed in panel (d) is close to the theoretical maximum value of 2.77 [64].

**Figure 6:**
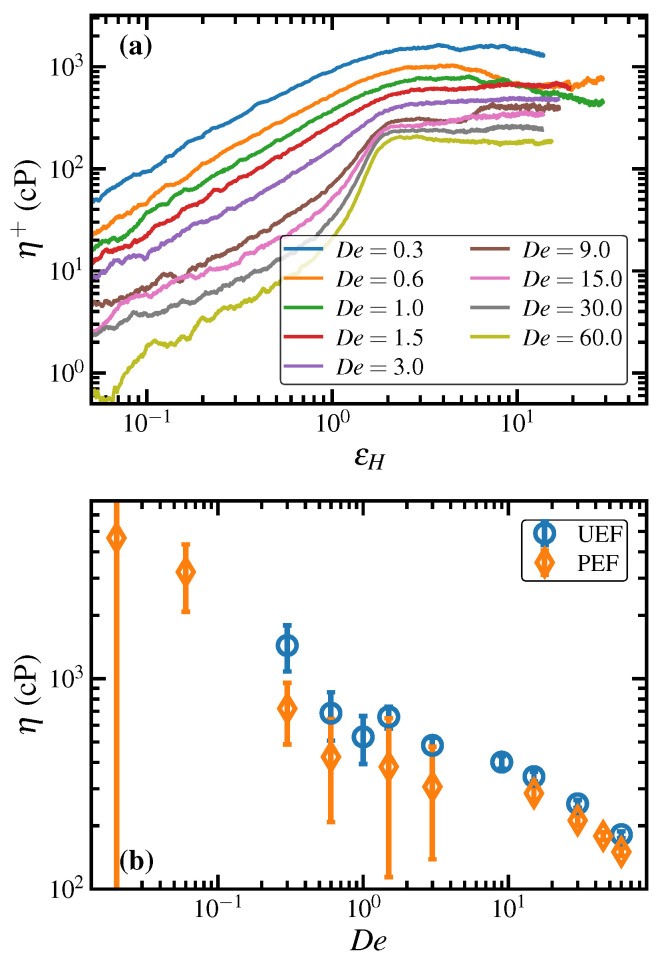
Startup ((**a**); UEF only) and steady-state ((**b**); both UEF and PEF) viscosity for the PE melt undergoing elongational flow at various De. Statistical error bars are indicated for the steady-state data displayed in panel (**b**).

The elongational viscosity upon startup ($\eta^{+}$) of UEF at various De is displayed in Figure 6a as a function of applied Hencky strain. At low values of De(≤1.0), a rapid initial increase in $\eta^{+}$ eventually leads to a maximum that gradually fades to a steady-state plateau at large $\varepsilon_{H}$. At all larger De, the overshoot is not apparent, with the initial increase eventually plateauing at its steady-state value. The sole exception is the case of De=9.0, which is at the lower limit of the FIC range of De. In this case, there is an initial plateau that appears in $\eta^{+}$ at $\varepsilon_{H}\approx 2$, and then a rapid increase to a larger value of $\eta^{+}$ at $\varepsilon_{H}\approx 6$. This behavior is consistent with that discussed above with reference to the molecular mean fractional extension evident upon startup of UEF at De=9.0 in Figure 1a (as well as the bottom right panel of Figure 2) and is associated with the initial formation of a stretched liquid phase upon startup of flow, which eventually transitions into a semicrystalline state after the molecules have become sufficiently stretched and aligned along the flow direction.

Steady-state values of the elongational viscosity are displayed in Figure 6b for both UEF and PEF. These quantities are defined as

(8) $$\eta =\frac{\sigma_{11}-1/2\;\sigma_{22}-1/2\;\sigma_{33}}{3\dot{\varepsilon }}\;\;\;\mathrm{for}\mathrm{UEF}\;\mathrm{and}\;\;\;\eta_{1}=\frac{\sigma_{11}-\sigma_{22}}{4\dot{\varepsilon }}\;\;\;\mathrm{for}\mathrm{PEF}\;\;,$$

where $\sigma_{ii}$ is a diagonal component of the Irving-Kirkwood stress tensor [72] with the 1-component denoting the flow direction. At low De and within the biphasic region, the PEF data are subject to large statistical error because of the small size of these simulations; however, the much larger UEF simulations have much greater statistical certainty. At low De (<0.1) (where only PEF data are available), the viscosity is consistent with a linear viscoelastic behavior exhibiting a constant viscosity, within statistical limitations. At intermediate De (0.3–1.5) within the biphasic region, however, the viscosity appears to decrease to a plateau value, and then decreases again at higher De (>3.0). The steady-state viscosity data of the PE melt under UEF and PEF evidently overlap, within statistical uncertainty, over the entire range of simulated De. This observation is quite remarkable physically, and it also speaks to the consistency of the simulations since the PEF simulations were drastically smaller in size than the UEF simulations, and given the fact that the PEF simulations at De<15.0 were performed in the NVT ensemble, whereas all UEF simulations were performed in the NpT ensemble. Furthermore, the PEF simulations were performed with KRBC, whereas the UEF simulations implemented the DHBC.

**Figure 7:**
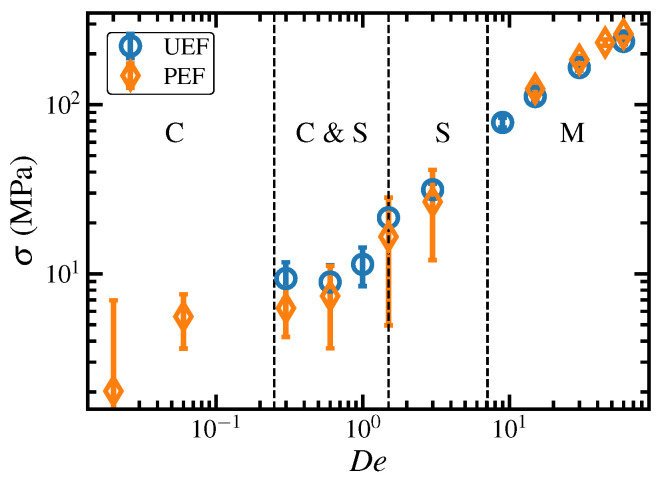
Nonequilibrium phase diagram of the $C_{1000}H_{2002}$ melt under both steady-state UEF and PEF based on the extensional stress, σ, plotted vs. De. Upper-case Roman letters within the figure are described in the caption to Figure 1.

The steady-state elongational stress, $\sigma \equiv \sigma_{11}-\sigma_{22}$ for PEF and $\sigma \equiv \sigma_{11}-\sigma_{22}/2-\sigma_{33}/2$ for UEF (where the index 1 is associated with the flow direction), is plotted versus De in Figure 7 for both UEF and PEF. Again, the results of both types of flow simulations appear to overlap to within statistical error. As expected, the stress increases monotonically at low De where only a coiled liquid phase exists (C), but within the biphasic region (C & S) where coiled and stretched liquid states coexist, the trend is not definitive. At higher De (> 1.5), however, the trend is once again monotonically increasing (S), even throughout the semicrystalline FIC regime (M).

#### 3.1.2. Flow-Induced Crystallization at High Deborah Number

Typical semicrystalline polyethylenes have normal melting points that vary greatly from low density to high density materials (0.88–0.96 $g/{\mathrm{cm}}^{3}$), ranging roughly from 385–420 K for common varieties. The heat of fusion, $\Delta H_{f}$, is generally accepted at roughly 290 J/g [73,74,75], and many studies indicate that single crystals formed from a quiescent melt assume orthorhombic or hexagonal lattices [46,76], whereas crystals formed under application of a mechanical tension tend to arrange into a monoclinic structure [77,78,79,80]. Indeed, a wide array of interesting semicrystalline morphologies can be generated under the application of a flow field, including the “shish-kebab” fibrillar structure of transverse lamellae emanating from a central axial fibril [81,82,83], at temperatures several decades above the quiescent melting point [24,84].

Toda [85] observed a melting point ($T_{m}$) of 398–403 K for single crystals of a linear PE of molar mass 13,000 g/mol grown under quiescent conditions. This is very close to the molar mass of the linear $C_{1000}H_{2002}$ melt (14,002 g/mol) simulated in this study. To date, it has been unfeasible computationally to simulate quiescent crystallization of entangled polyethylenes to determine a precise value of $T_{m}$ from molecular dynamics simulations, but the accrued evidence suggests a reasonable range would be 385–400 K [28,32,33,59,86]. Therefore, the simulated melt temperature employed in the present simulations is likely greater than 50 K than the value of $T_{m}$ determined via experiment or equilibrium MD simulation.

The observed discontinuities in fractional extension profiles at high De≥9.0 in Figure 1a, as well as the two-stage behavior at De=9.0 evidenced in the same figure, and also Figure 2 (bottom right panel) and Figure 6a at $3\leq \varepsilon_{H}\leq 6$, serve to indicate the possibility of a phase transition occurring within the PE liquid after an initial rapid formation of a highly stretched homogeneous liquid state. In fact, prior work has demonstrated that $C_{1000}H_{2002}$ undergoes flow-induced crystallization when subjected to strong PEF at De≥15 at a temperature of 450 K [17,18]. In this subsection, the FIC phase produced under UEF is examined in detail and compared with the same FIC phase developed under PEF at comparable De.

A variety of mathematical variables have been used to distinguish between liquid and crystalline phases in molecular simulations, as well as to quantify the degree of order induced in the FIC phase. These quantities aim to estimate the global or local ordering of polymer molecules or constituent segments within the simulation cell. One of the most common ones is the order parameter defined as $q\equiv \frac{3}{2}\lambda$, where λ is the eigenvalue of the segmental order parameter tensor, Q, with the largest absolute value. The segmental order parameter tensor is defined herein as

(9) $$Q\equiv \langle t_{i}t_{i}\rangle -\frac{1}{3}I\;\;,$$

where $t_{i}$ is a unit vector connecting adjacent even-numbered and odd-numbered carbon atoms along the chain backbone (which are separated by a single monomeric unit). The brackets 〈·〉 denote an ensemble average over all unit vectors, and I is the unit tensor. This definition and similar order parameters, which describe an ensemble average of the local degree of ordering within the sample, are somewhat arbitrary and are usually made based on *a priori* assumptions about the state of the polymeric material obtained from the simulation at specific state points. For example, Nafar Sefiddashti et al. [16,17,18] used the ranges q>0.75 to define a semicrystalline state, 0.65<q<0.75 for an ordered pretransitional liquid state, 0.25<q<0.65 for a stretched liquid, and q<0.15 for a coiled liquid; however, these points were defined rather arbitrarily from a preconstructed nonequilibrium phase diagram [17].

**Figure 8:**
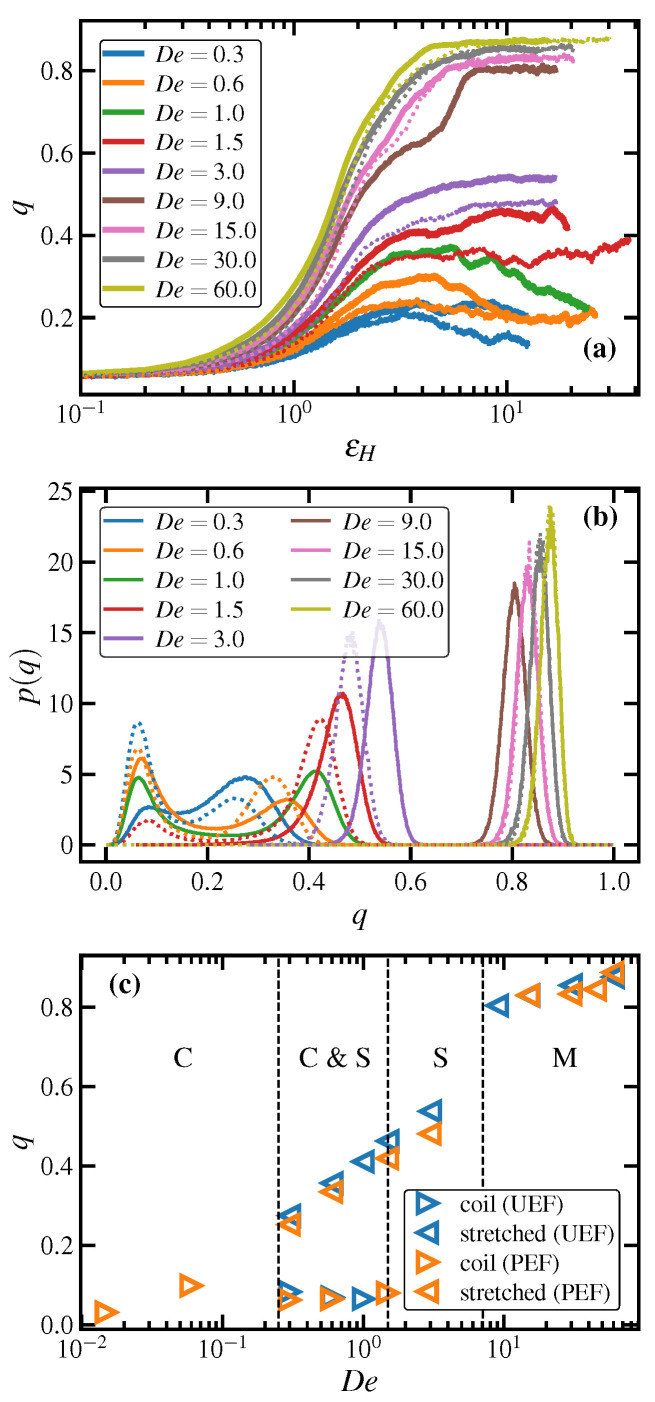
(**a**) Transient order parameter, *q*, as a function of Hencky strain, $\varepsilon_{H}$, at various values of De for startup of UEF and PEF of the PE melt at 450 K. (**b**) Steady-state probability distribution functions, p(q), of the order parameter at various values of De under UEF and PEF. (**c**) Steady-state order parameter vs. De for both UEF and PEF simulations. Vertical dashed lines define approximate boundaries of the various states or phases. Symbols C, S, and M denote coiled liquid, stretched liquid, and monoclinic semicrystalline states, respectively. The solid curves in panels (**a**,**b**) depict UEF data, whereas the dashed lines represent PEF data. Note that De=1.0 and 9.0 were not simulated under PEF, and De=0.015, 0.06, and 45.0 were not simulated under UEF.

The transient startup behavior of the order parameter is displayed in Figure 8a as a function of Hencky strain for both UEF and PEF at various De. These data are qualitatively similar to their counterparts in Figure 1a for the mean fractional extension. Specifically, the same three flow regions described in Section 3.1.1 are noticeable here as well. Characteristic overshoots and pronounced long-lived fluctuations are evident within the biphasic range 0.3≤De<1.5, followed by smoothly and monotonically ascending *q* within the range 1.5≤De<9.0, with *q* values indicating a stretched liquid state. At De≥9.0, a discontinuity is evident as the steady-state values of the curves jump significantly from those at lower De, similarly to the plots of 〈x〉 in Figure 1a. All De≥9.0 display steady-state values greater than the critical value of q=0.75 that indicates a transition from a stretched liquid state to a semicrystalline one.

The startup behavior of *q* at De=9.0 is a special case that requires additional deliberation. Indeed, De=9.0 is the critical flow strength at the lower limit of the transition from the stretched liquid phase to the semicrystalline phase. This transition is tracked by observing the evolution of the order parameter, which initially increases with $\varepsilon_{H}$ in a similar fashion as those of De=1.5 and De=3.0. Specifically, *q* exhibits a stretched liquid-like response by approaching a steady-state value of about q≈0.6, well below the critical order parameter of 0.75, up to a Hencky strain of $\varepsilon_{H}\approx 3$. At this point, the *q* curve passes through an inflection point and rises at a high rate for nearly two Hencky strains until it attains a new steady-state of roughly q≈0.8. Note that for PEF, a similar qualitative behavior with an inflection point at $\varepsilon_{H}\approx 2$ was observed at De=15.0, which was the lower limit of the FIC regime under PEF noted in Ref. [18]. This provides an indication that UEF is a stronger flow for orienting and extending the chain-like molecules than PEF due to the simultaneous compression along the two perpendicular axes with respect to the flow direction under UEF.

The steady-state PDFs of the order parameter (see Figure 8b) of the PE melt under both UEF and PEF are bimodal in the first De (< 1.5) region, with a low-*q* peak at q≈0.075 corresponding to essentially random coils and a high-*q* peak at q≈0.25–0.4, corresponding to moderately aligned and stretched chains. The high-*q* peak position shifts to the right (i.e., higher *q* values) as De increases. Within the range 1.5≤De<9.0, the distributions become unimodal and approximately Gaussian, varying roughly within the range 0.25<q<0.65, which is consistent with a purely stretched liquid state. At higher De, the PDFs significantly shift to the right and become narrower at *q* values greater than 0.75, demonstrating a highly ordered semicrystalline phase. Although differences between UEF and PEF in the PDFs of 〈x〉 are difficult to discern in Figure 1c, the PDFs of *q* are more clearly evident, where it is obvious that UEF is a stronger flow, producing the same effects as PEF but at slightly lower De. For instance, the biphasic region for UEF occurs within the range 0.3≤De≤1.0, whereas for PEF it occurs at 0.3≤De≤1.5. Furthermore, the FIC region begins at De=9.0 under UEF but at De=15.0 under PEF [18], although once inside the FIC regime, the PDFs of UEF and PEF practically coincide.

The peak positions of the p(q) are plotted as functions of De in Figure 8c. Note that outside the biphasic region (C & S), the peak positions and the ensemble averages are virtually identical. This figure is very similar to that of fractional extension displayed in Figure 1f, except that the distinction between the liquid and semicrystalline phases is significantly more pronounced here. This plot effectively serves as a nonequilibrium phase diagram of the PE melt under elongational flow, and a hysteresis loop for the $C_{1000}H_{2002}$ liquid in the biphasic region is evident at the constant temperature of 450 K, rendering this De region as a possibly universal feature of extensional flow.

**Figure 9:**
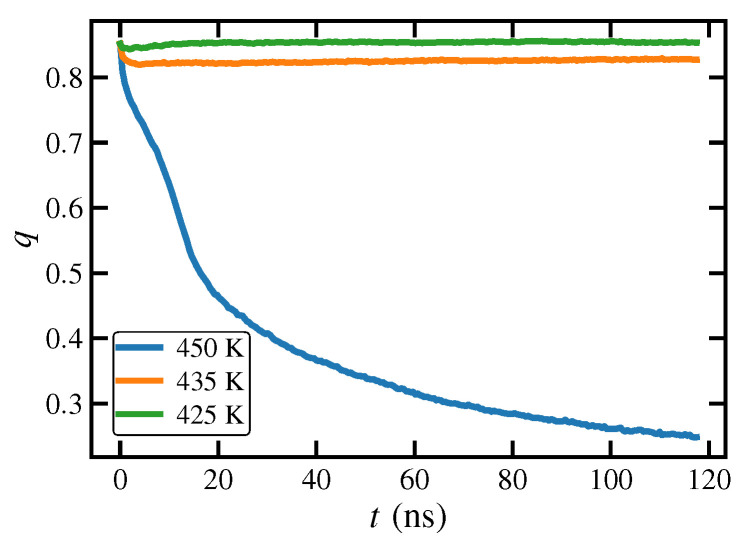
Transient order parameter *q* vs. time, *t*, after cessation of steady-state UEF at De=30.0 of the PE melt at 450 K with and without simultaneous quenching to a lower temperature, as labelled in the legend.

A plot of *q* versus time after cessation of steady-state UEF at De=30.0 and 450 K is presented as Figure 9. In these virtual experiments, the temperature can be quenched at the moment that the flow is turned off. If temperature is held fixed at 450 K, *q* rapidly relaxes back to its quiescent value associated with a liquid composed of coiled macromolecules, indicating melting of the semicrystalline phase that was formed under flow. Interestingly, if the temperature is simultaneously quenched to 425 K or 435 K at the moment of flow cessation, *q* remains essentially fixed at the same steady-state value (≈0.83), indicating that the semicrystalline phase remains indefinitely. This provides evidence that the semicrystalline phase formed under UEF has a melting point somewhere between 420–450 K, which is somewhat higher than the case for PEF of the same $C_{1000}H_{2002}$ liquid that melted between 415 K and 420 K in prior work [17]. Hence the FIC semicrystalline phase formed under UEF appears to be more stable than a similar FIC phase formed under PEF. The effective melting point is at least 35 K above the melting point of the quiescently grown crystal of 385–400 K, which implies a greater thermal stability of the FIC phase. This stability was examined by Baig and Edwards from the perspective of configurational temperature [28,29,87] and found that the FIC phase corresponded to a configurational state of the material, as imposed by the flow, that could be as much as several decades below the kinetic temperature. In other words, the configurational state adopted by the system in response to the flow implied a molecular ordering of the chains that was consistent with a much lower effective temperature, possibly even lower then the nominal melting point.

**Figure 10:**
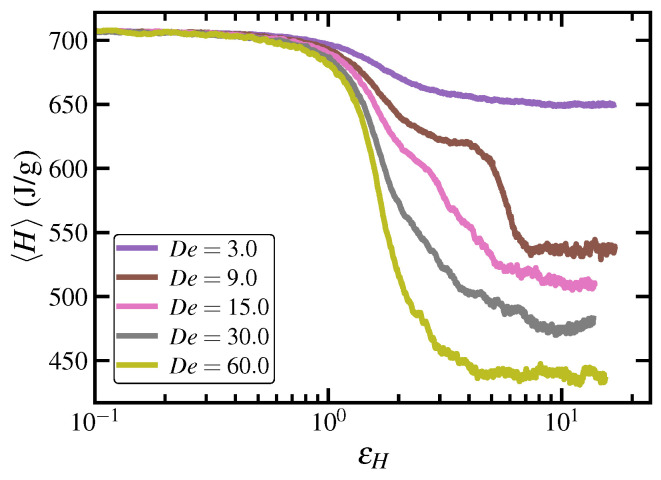
Transient ensemble-averaged enthalpy vs. Hencky strain upon startup of UEF of the PE melt at selected values of De. The precipitous drop of enthalpy at $\varepsilon_{H}\approx 4$ at De=9.0 corresponds to the steep increase of *q*, as depicted in Figure 8a, which underscores the onset of FIC at this value of Hencky strain.

The enthalpy change of the $C_{1000}H_{2002}$ melt under an applied UEF at several De is presented in Figure 10, where the enthalpy was calculated as $\langle H\rangle =\langle E_{tot}\rangle +\langle pV\rangle$ and $\langle E_{tot}\rangle$ is the ensemble-averaged sum of the potential and kinetic energies. At De=3.0 (and similarly for lower values), the enthalpy gradually decreases upon startup of flow, but this descent accelerates after one Hencky strain unit of deformation and eventually settles into a lower enthalpy state associated with the stretched liquid phase. This decrease in 〈H〉 is due to the changing configuration of the chains from random coils within the quiescent melt to highly elongated and aligned molecules in the stretched liquid phase, which represents a more favorable energy state once entropic forces have been overcome by the applied flow. At higher De within the FIC regime, the rapid descent of enthalpy is much larger in magnitude before eventually levelling off to a steady-state value. Note that at De=9.0, the decrease in enthalpy follows a two-step process in which there is an initial decrease to a stretched liquid state ($\varepsilon_{H}<4$), followed by a further decrease to the FIC phase at $\varepsilon_{H}\approx 7$. Evidently, a sufficient degree of ordering and stretching of the macromolecules is necessary before the FIC phase can begin to develop. At De higher than 9.0, the ordering and alignment is sufficiently fast that the first step of the process blends in with the second. This is related to the formation and propagation of flow-enhanced nucleation events, which will be discussed later.

The heat of fusion of the semicrystalline phase grown under elongational flow can be estimated from the steady-state enthalpy at various De and compared to the quiescent value (≈707.7 J/g) according to $\Delta H=\langle H\left(De\right)\rangle -\langle H\left(0\right)\rangle =\Delta \langle E_{tot}\rangle +\Delta \langle pV\rangle$. Several steady-state values of enthalpy in the FIC region are computed as 〈H(15)〉=510.4 J/g, 〈H(30)〉=476.1 J/g, and 〈H(60)〉=439.0 J/g, which provide ΔH values of −197.3, −231.6, and −268.7 J/g, respectively. A similar value of −252 J/g was calculated at De=60.0 in the PEF simulations of Nafar Sefiddashti et al. [17]; note that this value was erroneously reported by the authors in Ref. [17] as −283.5 J/g. These values effectively quantify the amount of heat released upon stretching and packing the molecules into the semicrystalline phase. At De=9.0, the enthalpy change associated with the stretched liquid state comprises about 50% of the total steady-state ΔH. This indicates that a substantial amount of the imposed elongational force is required to deform and orient the macromolecules, which subsequently can more readily pack into the monoclinic lattices of the semicrystalline phase (with a commensurate density reduction), as discussed below. The reported values are of similar magnitude and range to experimental heats of fusion for typical (linear) PEs, which range from −250 to −290 J/g for PEs with degrees of crystallinity covering a wide range of values (roughly, 60–90%) [73,74,75,88].

**Figure 11:**
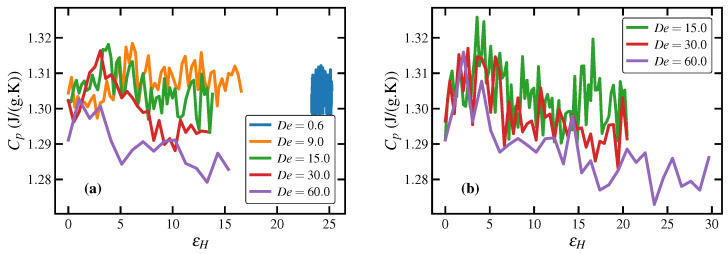
Specific heat capacity vs. Hencky strain of the PE melt under startup of UEF (**a**) and PEF (**b**) at various De. Note that data at De=0.6 in panel (**a**) were taken under steady-state conditions.

Specific heat capacity at constant pressure, $C_{p}$, is plotted versus Hencky strain in Figure 11 under startup of UEF (panel a) and PEF (panel b) at several values of De in the FIC regime. The quantity was calculated from the energy dispersion relationship used in NpT equilibrium molecular dynamics [89],

(10) $$C_{p}=\frac{\langle E_{tot}^{2}\rangle -{\langle E_{tot}\rangle }^{2}}{k_{B}T^{2}}\;\;,$$

although there is no explicit guarantee that this expression remains valid away from equilibrium. As evident from the figure, this quantity fluctuated markedly, although the *y*-axes in the panels are magnified to accentuate the differences between the various curves, which serves to exaggerate the actual magnitude of the fluctuation. At low $\varepsilon_{H}$, all curves emanate from the quiescent value of about 1.305 J/(g K). Note that for De less than 9.0, where the PE melt remains in a liquid state, $C_{p}$ retains its quiescent value even under nonequilibrium conditions, as illustrated in panel (a) for UEF at De=0.6. However, at De inside the FIC regime, $C_{p}$ decreases slightly upon startup of flow in both UEF and PEF, ultimately approaching steady-state plateaus with values that decrease with increasing De. Nevertheless, from the quiescent state to the steady-state value at De=60.0 (about 1.28 J/(g K)), the change in heat capacity is minimal (about 2%). Typical experimental values of specific heat capacities for polyethylenes generally range from 1.3–2.7 J/(g K) [88,90,91,92] at temperatures up to about 450 K. Hence the values computed from the NEMD simulations are at the low end of the range.

Ensemble-averaged measures of order, such as 〈x〉 and *q*, cannot provide any information concerning the heterogeneous, localized microstructure development that occurs under application of elongational flow. Recently, local thermodynamic-like variables at the atomistic level, such as entropy and enthalpy, were introduced as a means of quantifying the spatial and temporal evolution of flow-enhanced nucleation that spontaneously occurred in the $C_{1000}H_{2002}$ melt under PEF at 450 K [16]. The advantage of these variables is that they are associated with each atom in the simulation, defined within a very small environment of that particular particle. They are thus defined based on very localized spatial averages, but no temporal averaging is applied. This implies that these variables can change from one time step to the next, and their time evolution can thus be tracked, rendering an efficient mechanism for observing flow-enhanced nucleation (FEN) events as they appear and grow in size or wane and fade away over the course of a simulation. A key aspect of these variables is their ability to distinguish between small liquid-like and crystalline-like regions within the bulk sample. Of course, these variables can also be ensemble-averaged, if desired, to provide a gross measure of the macroscopic entropy and enthalpy of the bulk liquid.

The local or atomistic entropy, $S_{i}$, is estimated from the atomic radial distribution (pair correlation) function defined relative to particle *i* according to

(11) $$S_{i}=-2\pi \rho_{N}k_{B}\int_{0}^{r_{m}}\left[g^{i}\left(r\right)\ln g^{i}\left(r\right)-g^{i}\left(r\right)+1\right]r^{2}\;dr\;\;,$$

where $\rho_{N}$ (=2.0 in LJ units for the quiescent liquid) is the overall particle number density, $k_{B}$ is Boltzmann’s constant, *r* is the spatial coordinate, and $r_{m}=20$ Å (5.1 in LJ units) is the cut-off limit for the integration [18]. $g^{i}\left(r\right)$ is the radial distribution function (RDF) centered at the *i*-th atom. (Note that the effect of particle density on atomic entropy was discussed extensively in Appendix A of Ref. [18]. The effects of the other parameters were analyzed in Appendix B of Ref. [18]. In addition, note that $\rho_{N}\approx 2.0$ in LJ units for the liquid state and higher for the semicrystalline state; its value was adjusted in accordance with the NpT simulations). The RDF is approximated as

(12) $$g_{m}^{i}\left(r\right)=\frac{1}{4\pi \rho_{N}r^{2}}\sum_{j}\frac{1}{\sqrt{2\pi \sigma^{2}}}e^{-{\left(r-r_{ij}\right)}^{2}/\left(2\sigma^{2}\right)}\;\;,$$

where $r_{ij}$ is the distance between atoms *i* and *j* (i.e., the neighbors of atom *i*) and σ (=0.07 in LJ units) is a broadening parameter [18]. The average local atomistic entropy, ${\bar{S}}_{i}$, is defined as

(13) $${\bar{S}}_{i}=\frac{\sum_{j}S_{j}+S_{i}}{N_{neigh}+1}\;\;,$$

and it is used herein in lieu of $S_{i}$ to increase the resolution and continuity in distinguishing between different highly localized phases. In this expression, $N_{neigh}$ is the number of neighbors of atom *i* within a cut-off distance of $r_{c}=9.83$ Å (or $2.5\sigma_{CH_{2}}$). One advantage of $S_{i}$ and ${\bar{S}}_{i}$ over other measures of entropy is that they are defined only over a small neighborhood of a given particle, not ensemble-averaged over all particles within the simulation cell [18]. Therefore, they can distinguish local regions of liquid-like or solid-like behavior that coexist within an inhomogeneous sample. This can be a great aid to examining flow-enhanced nucleation and flow-induced crystallization, as discussed below.

The local or atomistic enthalpy is defined as $H_{i}=E_{i}+\left(pV\right)/N$, where *p* and *V* are the system pressure and volume, respectively, and *N* is the total number of particles. (An extensive discussion of this definition was provided in Appendix A of Ref. [18]). $E_{i}$ is the potential energy of atom *i*, which is readily calculated from the simulation output. The average local enthalpy, ${\bar{H}}_{i}$, is defined in a similar manner as the corresponding average local entropy, $\bar{S_{i}}$ in Equation (13). It should be noted that the atomistic entropy and enthalpy were first introduced and used by Piaggi et al. [93] to distinguish between liquid and various solid crystalline phases of sodium and aluminium in molecular dynamics simulations. See Refs. [18,93] for additional details of the method and analysis of the numerical values of the model parameters in Equations (11)–(13).

By examining the 3-dimensional probability distribution functions of ${\bar{S}}_{i}$ and ${\bar{H}}_{i}$ for liquid and semicrystalline phases of the $C_{1000}H_{2002}$ melt at 450 K (9.5745 in LJ units), the liquid-to-crystalline state threshold values for the average local entropy, ${\bar{S}}_{i}^{th}$, and enthalpy, ${\bar{H}}_{i}^{th}$, were determined as ${\bar{S}}_{i}^{th}=-5.8$ and ${\bar{H}}_{i}^{th}=7.0$, both expressed in reduced LJ units— see Figure 2 of Ref. [18]. In other words, particles occupying liquid-like local locations most likely possess a local entropy greater than −5.8 and a local enthalpy greater than 7.0, whereas the local entropy and enthalpy for particles in crystal-like neighborhoods would be less than −5.8 and less than 7.0, respectively. These threshold values provide a simple and direct criterion for distinguishing liquid and crystalline phases in MD simulations of polyethylene melts [18].

The local thermodynamic-like variables defined above can be ensemble averaged (with respect to space and time) to provide some bulk-scale measures of the atomistic energetics and local ordering of the PE melt under elongational flow. Note, however, that these quantities should not be interpreted as inherent thermodynamic properties of the fluid. For instance, the long-range configurational entropy of the polymer chains is not accounted for in the atomistic entropy expression for $S_{i}$, nor the kinetic energy of the atoms included in the atomistic enthalpy calculation, and so on. Rather, these variables are in essence microstructural variables, such as *q*, whose magnitude provides a rough measure of system order. The advantage of using these ensemble-averaged variables over *q* is that they possess a clear discontinuity at phase transitions, which is completely lacking in *q*.

**Figure 12:**
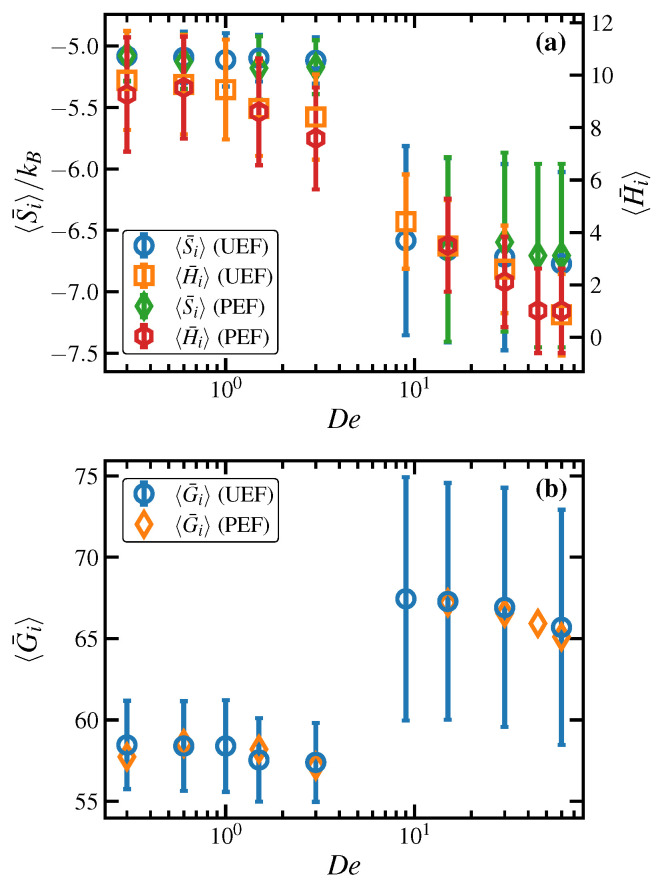
The ensemble-average local atomic entropy, enthalpy (panel (**a**)), and Gibbs free energy (panel (**b**)) as functions of De for the UEF and PEF simulations of the $C_{1000}H_{2002}$ melt in (dimensionless) reduced LJ units. Note the high degree of agreement between UEF and PEF data, in spite of the large error bars on some of the data.

Figure 12a displays the steady-state ensemble-averaged local atomistic entropy, $\langle \bar{S_{i}}\rangle$, and enthalpy, $\langle \bar{H_{i}}\rangle$, as functions of De for the PE melt under both UEF and PEF. Both quantities practically overlap for the UEF and PEF simulations, suggesting a minor difference between uniaxial and planar extension concerning the short-range or local configurational properties and energetic responses of entangled linear melts to these purely extensional flow fields. The average local entropies are practically independent of the flow strength in the range 0≤De<9, but exhibit a dramatic discontinuous drop at De=9, again remaining constant at higher flow strengths. This discontinuity arises from a first-order type transition of a liquid to semicrystalline phase induced by the extensional flow. It is evident that the local ordering of the polymer is solely a function of the material phase and does not change with the flow strength and long-range orientation and stretching of the molecules. The local enthalpy behaves somewhat similarly in the sense that it exhibits a discontinuous drop at De=9 where the phase transition occurs. However, unlike $\langle \bar{S_{i}}\rangle$, $\langle \bar{H_{i}}\rangle$ is a relatively weak decreasing function of De in the liquid phase and a moderate descending function in the semicrystalline phase. This decreasing trend is attributed to the intermolecular LJ and torsional energies that continuously decrease even at very high De as molecules stretch and the inherent dihedral angles assume more *trans* conformations, which consequently, allow segments of individual chains to approach each other and pack into definite crystal lattices, thereby reducing the intermolecular LJ energy—see also Figure 11b of Ref. [18].

Inspired by a thermodynamic Legendre transformation, an average local Gibbs free energy can be defined as $\bar{G_{i}}=\bar{H_{i}}-T\bar{S_{i}}$. Figure 12b depicts the ensemble-average local atomic Gibbs free energy, $\langle \bar{G_{i}}\rangle$, for UEF and PEF as a function of De. Despite the large error bars, it is evident from this figure that the liquid and semicrystalline phases possess very different local Gibbs free energies, rendering this quantity a valuable phase assessment tool. Note that at high temperatures (such as 450 K), $\langle \bar{G_{i}}\rangle$ is dominated by the local entropy and seemingly not particularly informative; however, it was demonstrated previously [18] that the transient $\langle \bar{G_{i}}\rangle$ response exhibits a minimum at the initiation of FIC, which is an indication of FIC not manifested by either $\langle \bar{S_{i}}\rangle$ or $\langle \bar{H_{i}}\rangle$ individually. Using the stated values of ${\bar{S}}_{i}^{th}=-5.8$ and ${\bar{H}}_{i}^{th}=7.0$, and the dimensionless temperature corresponding to 450 K (9.5745), the threshold value of atomic Gibbs free energy is $\langle {\bar{G_{i}}}^{th}\rangle =62.5$ in LJ units. This quantity then provides a rather definitive value for distinguishing between liquid-like and solid-like behavior of the PE material, which can be exploited in the transient response of the melt under elongational flow.

**Figure 13:**
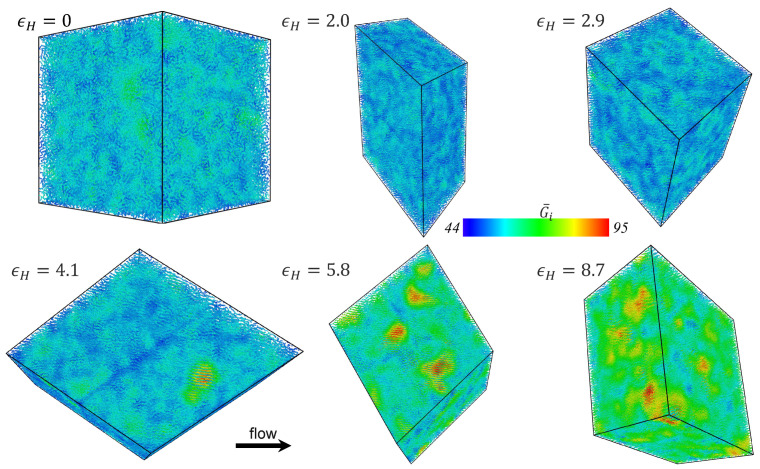
Snapshots of the simulation cell at various Hencky strains after startup of UEF at 450 K of the PE melt at De=9.0. Individual atoms are colored based on their instantaneous values of ${\bar{G}}_{i}$, according to the legend color bar. Note that the boxes are displayed at random angles (and sizes) relative to the flow direction because the simulation cell rotates (and elongates/compresses) with time under UEF due to the imposed boundary conditions.

To illustrate the use of local Gibbs energy in determining the state of the local microstructure in the PE material, Figure 13 displays snapshots of the simulation cell at various Hencky strain units for startup of UEF at De=9.0. The atoms comprising the simulation box are colored in the instantaneous snapshots based on their individual values of ${\bar{G}}_{i}$, with cooler colors representing low values of ${\bar{G}}_{i}$ and the opposite for warmer colors. Under quiescent conditions ($\varepsilon_{H}=0$), almost all simulation atoms have ${\bar{G}}_{i}$ values below the threshold value of ${\bar{G_{i}}}^{th}=62.5$; however, there are a number of randomly-located localized regions (of roughly 5–10 Å in size) that possess ${\bar{G_{i}}}^{th}>62.5$, indicating a degree of crystal-like ordering. These nucleation sites tend to appear spontaneously for a brief period of time and then gradually fade away, like fireflies flashing over a grassy field at dusk. At $0<\varepsilon_{H}<4$ after inception of flow, these crystal-like nucleation sites continue to appear spontaneously and then fade away at random locations under flow, but in greater number and possibly a larger overall dimension. (To date, work continues on a viable method to quantify the size and kinetics of this flow-enhanced nucleation.) At $4<\varepsilon_{H}<6$, the nucleates stabilize and begin to increase in size rapidly, ultimately forming into relatively large crystallites on the order of 50 Å in diameter.

We hypothesize that the onset of the phase transition behavior at $\varepsilon_{H}\approx 4$ of the De=9.0 simulation discussed above originates from random (with respect to space and time) flow-enhanced nucleation events of local crystal-like order that appear within small localized regions of the sample. At De<9.0, these fluctuating nucleates are unstable, possessing very short lifetimes, and disappear rapidly after formation before they can stabilize into localized semicrystalline regions. It is worth emphasizing that these random FEN events occur at all flow strengths, even under quiescent conditions (see the first snapshot at $\varepsilon_{h}=0$ of Figure 13). At higher De(≥9.0), once a critical value of strain deformation has been attained ($\varepsilon_{H}>4$ for De=9.0, but around two strain units for higher De), these nucleates stabilize and begin to grow in size, developing into stable crystalline domains that eventually enlarge to a global steady-state FIC phase (at $\varepsilon_{H}>6$).

An important aspect of FIC from both theoretical and practical perspectives is the kinetics of nucleation and crystallization. The threshold values of the local thermodynamic-like variables provide a means to delineate and quantify phase transitions in atomistic simulations. Since these quantities are computed for individual atomic units, they can be conveniently used to determine an average concentration or size for the liquid and semicrystalline phases at each time step of simulation. Such information conceptually facilitates the quantification of the dynamic process of crystallization (or melting), hence enabling measurement of the kinetics of the FIC process. Using these threshold values, the number of particles occupying liquid-like, $N_{l}$, and crystalline-like, $N_{c}$, neighborhoods, can be calculated, respectively, as the number of particles whose $\bar{S_{i}}>{\bar{S}}_{i}^{th}$ and $\bar{S_{i}}<{\bar{S}}_{i}^{th}$. The fraction of atoms occupying liquid and semicrystalline states are given by $\tilde{N_{l}}=N_{l}/N_{tot}$ and $\tilde{N_{c}}=N_{c}/N_{tot}=1-\tilde{N_{l}}$, where $N_{tot}=N_{l}+N_{c}$ is the total number of particles (united atoms) in the simulation cell.

In prior work [18], a first-order reversible reaction (L⇔S) between the liquid and semicrystalline states (or localized domains within the simulation cell) was proposed [18], and an expression was derived for quantifying the fraction of liquid-like particles within the simulation box as

(14) $$\tilde{N_{l}}\left(t\right)={\tilde{N}}_{l,ss}+\left[\tilde{N_{l}}\left(0\right)-{\tilde{N}}_{l,ss}\right]e^{-(k_{1}+k_{-1})t}\;\;,$$

where $k_{1}$ and $k_{-1}$ are the forward and reverse reaction rate constants with

(15) $${\tilde{N}}_{l,ss}=\frac{k_{-1}}{k_{1}+k_{-1}}=\frac{1}{K+1}\;\;,$$

where ${\tilde{N}}_{l,ss}$ is the fraction of atoms occupying local liquid-like environments at steady state and $K=k_{1}/k_{-1}$ is the equilibrium constant—see Ref. [18] for more details. The overall degree of crystallinity can be approximated as $\chi =1-{\tilde{N}}_{l,ss}=K/\left(K+1\right)$, which is essentially the steady-state fraction of particles occupying a semicrystalline local state.

**Figure 14:**
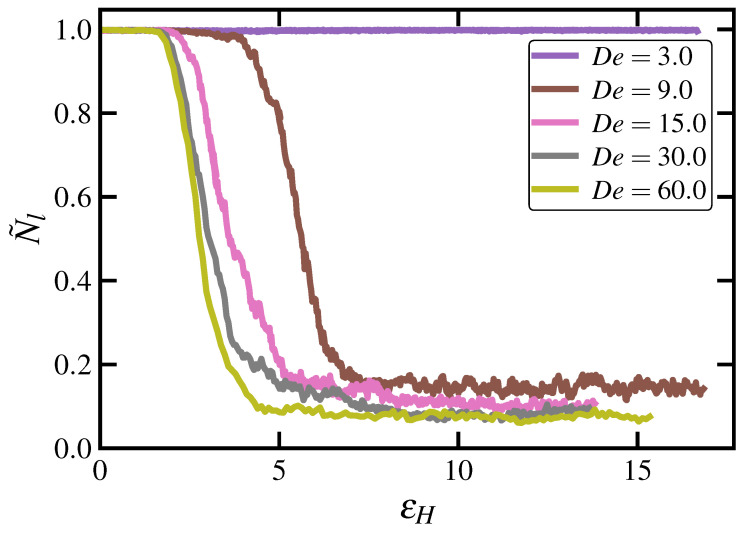
Fraction of liquid-like particles, ${\tilde{N}}_{l}$, as a function of Hencky strain within the PE material for startup of UEF at various De.

The fraction for the liquid-like phase upon startup of the $C_{1000}H_{2002}$ melt under UEF at various De is depicted in Figure 14 as a function of the Hencky strain (dimensionless time). It is apparent that the system remains liquid-like at De=3 with a value of ${\tilde{N}}_{l}\approx 1$ at all times, in agreement with all other evidence discussed in this section. At De=9, however, ${\tilde{N}}_{l}\approx 1$ only up to $\varepsilon_{H}\approx 4$, at which time the fraction of liquid-like particles begins to drop precipitously, plateauing at a low value of approximately 0.15 once steady state has been attained at $\varepsilon_{H}\approx 6$. The behavior of ${\tilde{N}}_{l}$ at higher De is qualitatively similar, although the onset of crystallization shifts to lower Hencky strains ranging from 2.0–2.5. These results are in agreement with the observed FIC onset in terms of $\varepsilon_{H}$ of prior PEF simulations [18]. The longer delay of FIC at De=9 arises from the fact that the dominant phase of the system at this critical flow strength is liquid up to $\varepsilon_{H}\approx 4$ when the crystallization initiated via flow-enhanced nucleation events (that are randomly located throughout the sample) begins to develop from stable nuclei—see Figure 8a, Figure 10 and Figure 13.

Table 2 summarizes the forward, $k_{1}$, and reverse, $k_{-1}$, rate constants obtained from a nonlinear least-squares fitting of $\tilde{N_{l}}$ data versus time (after excluding the initial lag times) to Equation (14) for De≥9 under steady-state UEF. The equilibrium constants, *K*, densities of the semicrystalline phase, $\rho_{c}$, and degrees (percentages) of crystallinity, χ, are also listed. The corresponding values from the PEF simulations (see Table 2 of Ref. [18]) are displayed in parentheses for comparison. It is evident from these values that the forward rate constants increase almost linearly with flow strength, implying a faster FIC at higher De. The reverse rate constants, equilibrium constants, and χ also increase with flow strength, although with significantly smaller slope values than that of $k_{1}$. Furthermore, the corresponding values of PEF agree reasonably well with the UEF values: the PEF rate constants are slightly higher than those of UEF at all De, however, their ratio, and therefore the equilibrium constants and degrees of crystallinity under PEF are slightly lower than the corresponding UEF values. Consequently, direct evidence once again indicates that UEF has stronger orientation and ordering capability than PEF. The values of $\rho_{c}$ and χ are quite reasonable based on observations from experiments of stretched PE samples [77,78,79,80].

**Figure 15:**
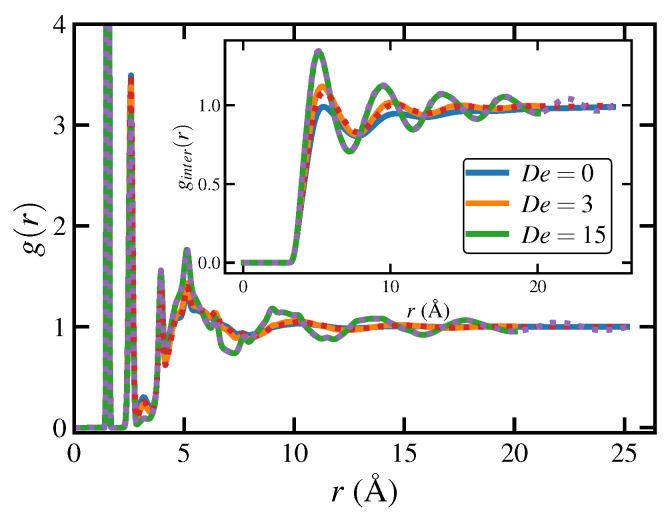
Steady-state radial distribution function, g(r), of the $C_{1000}H_{2002}$ melt at three values of De=0.0,3.0,15.0 under both UEF (solid lines) and PEF (dotted lines). Note that UEF and PEF data virtually overlap at all De; therefore, the PEF data curves are not labelled in the legend. The inset displays the intermolecular contribution to the overall RDFs displayed in the main graph.

The semicrystalline phase developed under flow via FIC using NEMD simulations can be characterized in terms of the radial distribution (pair correlation) function and structure factor, which allow direct comparison with spectroscopic experimental data. Figure 15 displays the total RDF, g(r), and intermolecular pair correlation function, $g_{inter}\left(r\right)$ (inset) of the $C_{1000}H_{2002}$ melt at equilibrium (De=0) and under UEF (solid lines) and PEF (dashed lines) for two representative extension rates, the lower (De=3) depicting a stretched liquid state and the larger (De=15.0) a semicrystalline phase. The first point to notice in this figure is the virtually identical curves of UEF and PEF at corresponding values of De, indicating negligible influence of the geometry of extensional flows on the short to medium length-scale ordering and microstructure of the molecules; this observation agrees very well with the overlapping curves of $\langle \bar{S_{i}}\rangle$, $\langle \bar{H_{i}}\rangle$, and $\langle \bar{G_{i}}\rangle$ observed in Figure 12.

As discussed by Nafar Sefiddashti et al. [18], there are five prominent peaks in each total RDF (g(r)) at positions 1.54, 2.58, 3.14, 4.04, and 5.16 Å (relative to the test atom at r=0), respectively denoting the bond distance (1,2 carbon atoms), bond angle (1,3 atoms), a *gauche* configuration, and *trans* configurations (1,4 and 1,5 atoms)—see Figure 9 of Ref. [41]. These peaks are present at all De, but the relative heights of the last three change with increasing De: the peak at 3.14 Å shrinks as the number of *gauche* dihedral angles diminishes as the molecules extend, whereas the last two peaks grow in height due to the increasing number of *trans* dihedral angles. At higher distances from the test atom (r>6Å), the equilibrium and De=3.0 distributions practically overlap since neither liquid possess any long-range order; however, at De=15.0, evident long-range ordering persists out to 20–25 Å.

The intermolecular distributions ($g_{inter}\left(r\right)$) at De=0,3.0 (shown in the inset of Figure 15) possess two distinct peaks at 5.46 and 10.34 Å, but practically no long-range order beyond the second nearest neighbor shell is observed. This suggests significant local longitudinal ordering of the stretched liquid-phase molecules along the flow direction at De=3.0, but very little lateral ordering of the individual chains perpendicular to their common axis of orientation. At De=15.0, however, the first two peaks of the intermolecular RDF shift slightly to the left at positions 5.10 and 9.48 Å, indicating a relatively higher density in the semicrystalline phase. The NpT ensemble simulations allow calculation of the density change due to crystallization, as the simulation cell dimensions are adjusted by the applied barostat. The simulation results show a 15–17.5% increase in the system density in the range 9.0≤De≤60.0 for the semicrystalline phase compared to the quiescent melt at 450 K, as tabulated in Table 2. Furthermore, the $g_{inter}\left(r\right)$ curve at De=15.0 has several additional peaks beyond the second one, indicating long-range intermolecular packing of the semicrystalline structure. These additional peaks decay after approximately 20–25 Å, indicating crystallites on the order of 50 Å in diameter. With the degree of crystallinity at roughly 90% at steady state, this implies that the various nucleation sites formed at small strains and random locations grow in size under continued strain and eventually encroach upon each other (see Figure 13), but do not merge together to form a single larger crystallite. It is therefore likely that there exist dislocation (and possibly disclination) defects lying between them.

**Figure 16:**
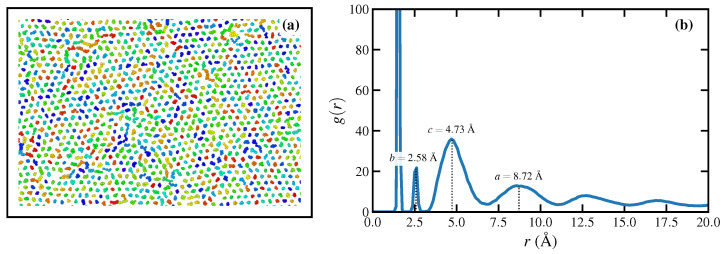
Panel (**a**): A snapshot of a random slab of thickness 11.8 Å and oriented perpendicular to the direction of flow of the semicrystalline PE taken under steady-state UEF at De=60.0. Individual molecules are assigned random colors, and each atom contained within the slab of a particular molecule is assigned the same color. Most molecules are aligned parallel to the flow direction and hence appear as large dots in the plane of the slab. Panel (**b**): A graph of g(r) for a random slab of thickness 3 Å oriented perpendicular to the flow direction, similar (but thinner than) the slab of Panel (**a**). The monoclinic lattice parameters are labelled in the plot.

The global ordering of the FIC state is depicted in Figure 16a for a random slab of width 11.8 Å (oriented perpendicularly to the direction of flow) of the PE semicrystalline phase produced under steady-state UEF at De=60.0. In this snapshot, taken at a random time step within the steady-state regime, atoms occupying different molecules have been assigned various assorted colors, whereas atoms belonging to the same molecule all possess the same color. Most of the molecules shown in the snapshot appear as single dots because the molecules are almost fully aligned axially, and the snapshot is perpendicular to the direction of alignment. Throughout the sample, semicrystalline order is apparent, interlaced with small regions of amorphous character.

The pair correlation function for a random slab of 3 Å width (oriented perpendicularly to the direction of flow) of the semicrystalline state at De=60.0 is displayed in Figure 16b. A very narrow slab was chosen to minimize the peak-broadening effects on g(r) from the axial (*z*) dimension; this allows for a much more accurate calculation of the crystal lattice parameters in the *x*–*y* plane, as discussed shortly. The first tall, narrow peak at 1.54 Å is associated with the carbon-carbon (1,2) bond distance. The second peak corresponds to the (1,3) carbon distance of 2.58 Å. The reason this peak is so small is because the slab is only 3 Å thick, and the molecules are mostly aligned axially such that these chain units are perpendicular to the slab; therefore, very few of these units lie within the slab chosen for the calculation. This distance represents the lattice vector *b*. The next two peaks are associated with lattice vectors *c* (4.73 Å) and *a* (8.72 Å), respectively. The fact that all three vectors are of unequal length, and since angles ∠ab and ∠bc are orthogonal whereas ∠ac is not, indicates a monoclinic lattice structure. Seto et al. [79] indexed a linear PE material formed under mechanical stress to a monoclinic lattice with a=8.09, b=2.53, and c=4.79 Å, which agrees reasonably well with the simulation results. The PEF simulations generated lattice parameters of a=8.68, b=2.58, and c=4.44 Å [17], which also agree well with the UEF and experimental values.

**Figure 17:**
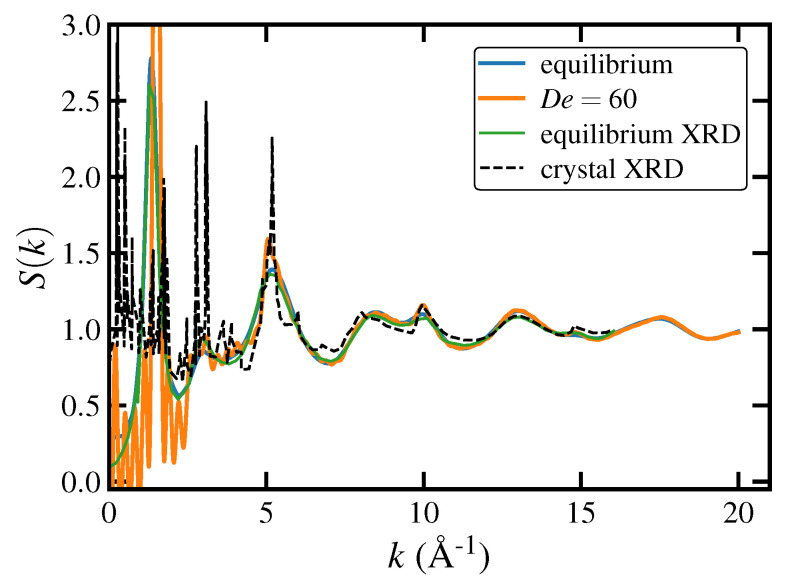
Steady-state structure factor, S(k), vs. wavenumber, *k*, of the $C_{1000}H_{2002}$ melt at De=0 and De=60 as calculated from the UEF simulations. In addition, there are the structure factors for an n-eicosane liquid (labelled as ‘equilibrium XRD’ in the legend) and solid crystal as obtained from X-ray diffraction experiments.

A method for characterizing and distinguishing liquid-like and crystal-like microstructures using MD data that offers direct comparison to experimental data is to calculate the static structure factor from the Fourier transform of the total radial distribution function, g(r). The structure factor can then be directly compared to experimental crystal X-ray diffraction (XRD) data. Figure 17 presents the structure factors for the simulated $C_{1000}H_{2002}$ melt at 450 K under equilibrium conditions (De=0.0; blue curve) and steady-state UEF at De=60.0 (orange curve). In addition, there are the structure factors determined using XRD experiments for a n-eicosane liquid at 315 K (green curve) and at 298 K for a quiescently grown crystal [27]. A comparison of the structure factors for the $C_{1000}H_{2002}$ melt at De=0 and 450 K and eicosane at 315 K reveals a direct overlap of the two curves at essentially all wavenumbers, one simulated and the other obtained from experiment. This provides reasonable evidence for the accuracy of the SKS potential model for alkane and PE materials. Both structure factors display characteristics of liquid alkane materials, as discussed elsewhere [27]. Of more importance, however, are the S(k) profiles for the simulated melt at De=60.0 and the experimental XRD data of the eicosane crystal, where the simulated PE exhibits distinct Bragg peaks at low wavenumbers that correspond closely to similar peaks in the experimental eicosane crystal, which was indexed to a triclinic crystal arrangement with lattice vector dimensions a=4.322Å, b=4.7999Å, and c=27.43Å, where the final value lies along the axis of the fully extended eicosane molecules constituting the crystalline material. The overall degree of similarity between the simulated and experimental S(k) curves provide additional evidence of the occurrence of FIC in the simulated PE melt under UEF. Note that a more detailed comparison between simulation and experimental *S*(k) can be found for PEF in Ref. [17].

### 3.2. The Role of Kuhn Segment Stretching in Flow-Induced Crystallization

**Figure 18:**
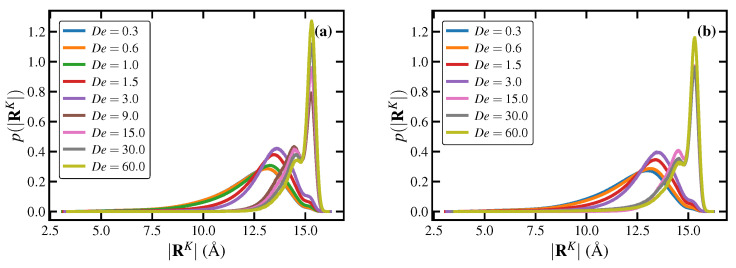
Probability distributions of the magnitude of Kuhn segments, $|R_{K}|$, comprising the PE melt under steady-state UEF (panel (**a**)) and PEF (panel (**b**)) at various De.

As mentioned in Section 2, the Kuhn segment length was calculated from the equilibrium simulation of the PE melt as 15.6 Å, comprised of 12 methylene units lying in a single plane, corresponding to all torsional dihedral angles occupying *trans* configurations. Hence the entire macromolecule consists of 83 Kuhn segments. However, plotting the probability distribution functions of the magnitude of the end-to-end vector of these segments, $|R_{K}|$, as displayed in Figure 18 at various De for both UEF (panel a) and PEF (panel b), revealed that the quoted length is actually the contour length of the Kuhn segments, not their end-to-end length, which takes on a rather wide distribution of magnitudes but rarely assumes the fully extended value of 15.6 Å. Even at intermediate De=0.3,0.6,1.0, these distributions essentially overlap the equilibrium PDF (not shown) with a mean of 11.8 Å and a peak at 12.8 Å, indicating that the Kuhn segments are significantly arced or bow-shaped. The distributions approach the null value on the left side around 8 Å, indicating the persistence length of the segments (which is roughly half the Kuhn length, as might have been expected), but they also approach the null value on the right side significantly below 15.6 Å, indicating the presence of essentially no fully extended segments. According to the SKS model, the torsional energy is minimized when all dihedral angles of the Kuhn segment are in the *trans* configuration (see Equation (6)), implying full extension, but the intramolecular LJ energy applies an attraction between carbon atoms within the segment separated by more than three bonds (see Equation (2)), which serves to collapse the segment into a bent structure.

As De increases into the stretched liquid state (De=1.5,3.0), the PDFs of the Kuhn segment magnitude shifts to the right and narrows, indicating that the segments are being extended by the flow. The effective persistence length, herein defined by the approach to the null value on the left side of the distribution, shifts higher to approximately 10 Å. These PDFs also tend to exhibit a small shoulder on the right side, indicating a slight fraction of fully extended Kuhn segments. At De where FIC has occurred (De≥9.0), however, the peaks of the PDFs become quite large and are positioned at the maximum segmental extension of 15.6 Å, indicating that the majority of segments are fully extended. There remains a small shoulder on the left side of the PDFs located at about 14.5 Å, which is associated with the existence of a single gauche dihedral angle at either end of the segment (see the bottom right stick diagram in Figure 9 of Ref. [41] and add 6×1.29 Å). The mean of these FIC distributions is 14.65 Å, and the effective persistence length has increased to approximately 12.5 Å. Over the entire range of De simulated, the PDFs under UEF and PEF essentially overlap each other at corresponding values of De.

In Figure 2 at De=9.0, the mean fractional extension begins to develop a steady-state value of 〈x〉≈0.85 at $2<\varepsilon_{H}<4$ corresponding to a stretched liquid state, followed by a rapid extension to the final steady-state value of 〈x〉≈0.95 over the range $4<\varepsilon_{H}<6$, which corresponds to an FIC phase. At a maximum extension length of 1290 Å, this difference amounts to 129 Å in molecular extension between the semicrystalline and stretched liquid phases, or 1.5 Å per Kuhn segment (based on 83 Kuhn segments per macromolecule). The peaks of the Kuhn segment PDFs in the stretched liquid state are observed at $|R_{K}|\approx 13.8$ Å. An additional 1.5 Å renders $|R_{K}|\approx 15.4$ Å, which is very nearly the maximum molecular extension. Hence this last bit of extension appears to be absolutely necessary to transition from the stretched liquid state to the semicrystalline phase.

**Figure 19:**
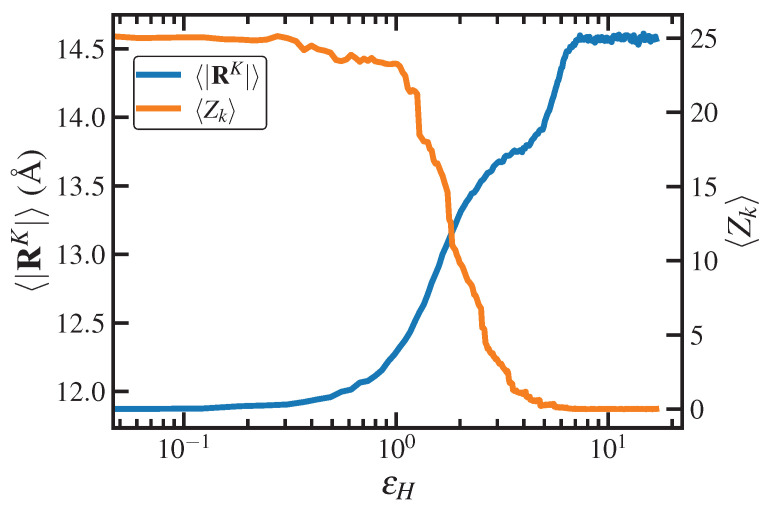
Mean value of the Kuhn segment magnitude, $\langle |R_{K}|\rangle$, and average number of chain kinks, $\langle Z_{k}\rangle$, vs. Hencky strain for startup of UEF at De=9.0.

A plot of the startup behavior of UEF at De=9.0 of the mean Kuhn segment magnitude, $\langle |R_{K}|\rangle$, and average number of chain kinks, $\langle Z_{k}\rangle$, as functions of Hencky strain units is displayed in Figure 19. The mean length of the Kuhn segments remains at its quiescent liquid value of 11.8 Å until $\varepsilon_{H}\approx 0.6$, after which it rises rapidly before approaching a steady-state plateau at 13.75 Å corresponding to a stretched liquid phase at $\varepsilon_{H}\approx 4$. During this same period of time, the entanglement network has been completely destroyed, as evident from the average number of chain kinks, which has decreased from its quiescent value of 24.3 to practically 0. At $\varepsilon_{H}=4$, the Kuhn segments begin to extend further while flow-induced crystallization is initiated, until the process attains a new steady-state at $\varepsilon_{H}\approx 6$ where the Kuhn segments are almost fully extended. It appears evident that 13.75 Å is a critical value of extension of the Kuhn segments before FIC can commence, and this value can only be achieved once the chain network is virtually completely disentangled, which occurs once the Kuhn segments of the individual molecules are all aligned along the flow direction end-to-end with a common axis. The additional extension (about 1.5 Å per segment, as mentioned above) of the Kuhn segments beyond the stretched liquid condition is apparently the primary driver of FIC, since all molecular extension within the range $4\leq \varepsilon_{H}\leq 6$ appears to be at the length scale of the Kuhn segments.

These results imply that flow-induced crystallization is not possible until the flow is strong enough to extend almost fully the majority of the Kuhn segments of the constituent chain-like macromolecules. This is physically sensible because crystallization occurs due to the lateral alignment of the extended molecules into local nucleates on the order of tens of Angstroms, and the lateral packing required to develop the observed monoclinic crystalline microstructures is only possible once the bow-like shape of the Kuhn segments has been almost fully straightened out. This appears to be possible once the entanglement network has been almost completely destroyed (which occurs at De=3.0 in the steady-state data of Figure 5a). Hence a sufficiently strong applied elongational flow that mandates an almost full extension of the Kuhn segments appears to be a prerequisite for FIC in this linear PE melt.

## 4. Conclusions

The atomistic simulations of the C_1000_H_2002_ melt undergoing steady-state and startup of uniaxial elongational flow reported herein complement those of prior simulations of the same polyethylene melt under planar elongational flow [7,8,17,18]. Indeed, most of the results regarding rheological, topological, and microstructural properties under both types of flow generally overlapped at corresponding values of De. This provided an indication of the universality of the observed properties since the different types of simulations, UEF and PEF, NVT and NpT, employed vastly different cell sizes and boundary conditions, yet still produced mutually consistent results at comparable Deborah numbers. Both types of flow generated a configurationally based microphase separation over similar De ranges, although the geometry of the bicontinuous phase was consistent with the geometry of the type of applied elongational flow. Both types of flow generated a flow-induced semicrystalline material at high De, with no apparent differences based on type of flow. However, the formation of the FIC phase was apparently predicated by the extension of the Kuhn segments constituting the polyethylene macromolecules. The FIC material possessed a nominal melting point roughly 35–40 K over the melting point of a quiescently formed crystal, and the heat of fusion and heat capacity of the simulations FIC phase compared favorably with experimentally determined values. Overall, the flow-induced phase separation and crystallization behavior was evidently a universal feature of elongational flows.

## Figures and Tables

**Table 1:** Quiescent properties of the simulated C_1000_H_2002_ melt. $\tau_{d}$ is the disengagement time, $\tau_{R}$ is the Rouse time, ${\langle R^{2}\rangle }^{1/2}$ is the average chain end-to-end distance, ${\langle R_{g}^{2}\rangle }^{1/2}$ is the average chain radius of gyration, 〈Z〉 is the average number of entanglements per chain, and $\langle Z_{k}\rangle$ is the average number of kinks per chain.

$\tau_{d}$ (ns)	$\tau_{R}$ (ns)	${\langle R^{2}\rangle }^{1/2}$ (Å)	${\langle R_{g}^{2}\rangle }^{1/2}$ (Å)	〈Z〉	$\langle Z_{k}\rangle$
5270	137	141.8	57.9	12.9	24.3

**Table 2:** Forward ($k_{1}$) and reverse ($k_{-1}$) kinetic rate constants at several De as calculated from the simulation output. Displayed are the corresponding equilibrium constant, $K=k_{1}/k_{-1}$, steady-state density of the semicrystalline phase, $\rho_{c}$, and degree of crystallinity, χ. Note that tabulated values in parentheses are the rate constants from the PEF simulations obtained from Ref. [18].

De	$k_{1}$ (ns^−1^)	$k_{-1}$ (ns^−1^)	*K*	$\rho_{c}$ ($g/{\mathrm{cm}}^{3}$)	χ (%)
9	0.0333	0.0047	7.0879	0.88	87.6
15	0.0532 (0.0634)	0.0048 (0.0069)	11.0 (9.14)	0.89 (0.91)	91.7 (90.1)
30	0.1194 (0.1497)	0.0087 (0.0149)	13.65 (10.05)	0.90 (0.91)	93.2 (91.0)
60	0.3264 (0.3467)	0.0228 (0.0354)	14.30 (9.80)	0.91 (0.91)	93.5 (90.7)

## Data Availability

The data that support the findings of this study are available on request from the corresponding author.
